# Alterations in lipidome profiles distinguish early-onset hyperuricemia, gout, and the effect of urate-lowering treatment

**DOI:** 10.1186/s13075-023-03204-6

**Published:** 2023-12-02

**Authors:** Aleš Kvasnička, David Friedecký, Radana Brumarová, Markéta Pavlíková, Kateřina Pavelcová, Jana Mašínová, Lenka Hasíková, Jakub Závada, Karel Pavelka, Pavel Ješina, Blanka Stibůrková

**Affiliations:** 1 Laboratory for Inherited Metabolic Disorders, Department of Clinical Biochemistry, University Hospital Olomouc and Faculty of Medicine and Dentistry, Palacký University Olomouc, Olomouc, Czech Republic; 2 Department of Probability and Mathematical Statistics, Faculty of Mathematics and Physics, Charles University, Prague, Czech Republic; 3 Institute of Rheumatology, Na Slupi 4, 128 50 Prague 2 Prague, Czech Republic; 4 Department of Pediatrics and Inherited Metabolic Disorders, First Faculty of Medicine, Charles University and General University Hospital, Prague, Czech Republic

**Keywords:** LC–MS, Lipidomics, Glycerophospholipids, Hyperuricemia, Gout, Urate-lowering treatment

## Abstract

**Background:**

Currently, it is not possible to predict whether patients with hyperuricemia (HUA) will develop gout and how this progression may be affected by urate-lowering treatment (ULT). Our study aimed to evaluate differences in plasma lipidome between patients with asymptomatic HUA detected ≤ 40 years (HUA ≤ 40) and > 40 years, gout patients with disease onset ≤ 40 years (Gout ≤ 40) and > 40 years, and normouricemic healthy controls (HC).

**Methods:**

Plasma samples were collected from 94 asymptomatic HUA (77% HUA ≤ 40) subjects, 196 gout patients (59% Gout ≤ 40), and 53 HC. A comprehensive targeted lipidomic analysis was performed to semi-quantify 608 lipids in plasma. Univariate and multivariate statistics and advanced visualizations were applied.

**Results:**

Both HUA and gout patients showed alterations in lipid profiles with the most significant upregulation of phosphatidylethanolamines and downregulation of lysophosphatidylcholine plasmalogens/plasmanyls. More profound changes were observed in HUA ≤ 40 and Gout ≤ 40 without ULT. Multivariate statistics differentiated HUA ≤ 40 and Gout ≤ 40 groups from HC with an overall accuracy of > 95%.

**Conclusion:**

Alterations in the lipidome of HUA and Gout patients show a significant impact on lipid metabolism. The most significant glycerophospholipid dysregulation was found in HUA ≤ 40 and Gout ≤ 40 patients, together with a correction of this imbalance with ULT.

**Supplementary Information:**

The online version contains supplementary material available at 10.1186/s13075-023-03204-6.

## Background

Gout is a prevalent form of inflammatory arthritis, with a rising incidence [1, 2], burdening individual health and impacting healthcare systems [3]. Reported estimates of gout prevalence range from 2.7 to 6.7% in countries with a Western lifestyle. The disease causes acute and intensely painful joint inflammation resulting from monosodium urate (MSU) crystal deposition and is associated with increased morbidity and mortality. The overall excess of mortality of 23 and 15% in men and women, respectively, is partially explained by an increase in cardiovascular diseases [4], renal disorders, digestive system diseases, and infections. Recently impaired cardiovascular and kidney functions have been more precisely described in gout patients [5], and interestingly, a neutrophil signature has been discovered [6]. According to the American College of Rheumatology guidelines, urate-lowering therapy/treatment (ULT) is not recommended for people with asymptomatic hyperuricemia. However, the potential of ULT for managing these “non-gout diseases” has been raised [7].

Published data shows that only one-third to one-half of patients with gout receive effective, curative ULT, and fewer than one-half of patients adhere to the prescribed treatment regimen. Recent data suggest that the number of patients presenting with gout under the age of 40 years (early-onset) is increasing [8]. These early-onset patients may have different clinical signs and comorbidities from those who present with gout at a later age [9, 10].

Hyperuricemia is a central feature in the pathogenesis of gout. Serum UA concentration presents a complex phenotype influenced by genetic and environmental factors and their interactions. The heritability of serum uric acid (UA) levels and gout has been estimated to be 27–41% worldwide and approximately 30% in Europeans [11]. The main cause of hyperuricemia is reduced renal excretion of UA. Accumulating evidence suggests that the net amount of excreted UA is regulated mainly by urate transporters, such as urate transporter 1 (a renal urate re-absorber) [12], solute carrier family 2 member 9 (*SLC2A9*, also known as glucose transporter member 9) [13, 14], and ATP-binding cassette subfamily G member 2 (*ABCG2*, a high capacity urate exporter expressed in the kidneys and intestines). Decreased extra-renal UA excretion, caused by ABCG2 dysfunction, is a common mechanism of hyperuricemia [15].

Gout progresses through several stages: asymptomatic hyperuricemia, acute gouty arthritis, intercritical gout, and chronic tophaceous gout. Not all individuals with hyperuricemia develop symptomatic gout, but the risk increases proportionally to the UA in the blood. Absent ULT, tophaceous gout usually develops ~ 10 years after the initial gout flare. The risk of gout development is conditioned not only on hyperuricemia but also on gender, weight, age, environmental and genetic factors, and their interactions. In some individuals with hyperuricemia or other major risk factors for gout, such as family history or MSU crystal deposition can be detected using imaging methods such as ultrasonography. Positive imaging findings are obtained in ~ 25% of individuals with hyperuricemia [16, 17], but it is currently unknown whether this predicts the development of clinically evident gout.

Many studies have pointed to an association between dyslipidemia, hyperuricemia, and gout. Dyslipidemia, characterized by total serum cholesterol, triacylglycerols, and low-density lipoprotein cholesterol (LDL-C), is positively associated with serum UA concentrations; in contrast, high-density lipoprotein cholesterol (HDL-C) is negatively associated [18]. Dyslipidemia is more frequently observed in patients with gout than in asymptomatic hyperuricemic patients [19], and patients with gout are more likely to have a history of dyslipidemia [20]. The dysregulation of lipids in hyperuricemia and gout has led to increased interest in understanding these associations relative to lipidomics, which can be used to profile hundreds of unique lipids in biological materials. Changes in glycerophospholipids and their lysoforms have been identified in rat models of potassium oxonate-induced gout [21]. Changes in triacylglycerols with predictive and diagnostic potential have been found in the serum of patients with hyperuricemia and gout [22]. Dysregulation of arachidonic and eicosapentaenoic-derived oxylipins, proposed as candidate serum biomarkers for differentiating gout from hyperuricemia, have been found in young men [23]. In terms of the molecular basis of these dysregulations, it has been shown in the HepG2 cell line and primary mouse hepatocytes that UA induces fat accumulation by stressing the endoplasmic reticulum and activating sterol regulatory element-binding protein-1c (SREBP-1c) [24]. Additionally, it has also been suggested that alteration in lysophosphatidylcholine metabolism during hyperuricemia might be caused by the upregulation of the lysophosphatidylcholine acyltransferase 3 enzyme (LPCAT3, EC 2.3.1.23) [25]. However, to date, an extensive lipidomic study (with large sample size) focusing on hyperuricemia and gout patients while considering early and late-onset and the effect of treatment has not been performed.

Our study aimed to evaluate the differences between the plasma lipidome profile of patients with asymptomatic hyperuricemia and gout versus normouricemic controls. Furthermore, we aim to characterize the changes in the lipidome between early and late asymptomatic hyperuricemia and gout (age ≤ vs. age > 40 years) and deepen our understanding of the molecular pathogenesis of gout, including the effect of ULT on plasma lipidome profiles.

## Methods

### Chemicals and reagents

Acetonitrile (ACN), isopropanol (IPA), water, and ammonium acetate (all LC–MS grade) were purchased from Sigma-Aldrich (St. Louis, MO, USA). SPLASH® LIPIDOMIX® Mass Spec Standard mixture and ceramide (d18:1-d7/15:0) and oleic acid-d9 (FA 18:1-d9) were purchased from Avanti Polar Lipids (Alabaster, AL, USA). Standard reference material NIST® SRM® 1950—“Metabolites in frozen human plasma” (SRM 1950) was purchased from Sigma-Aldrich (St. Louis, MO, USA).

### Patients

The analyzed set consisted of 94 patients with asymptomatic hyperuricemia (77% age detected ≤ 40 years) and a gout group of 196 patients (59% age detected ≤ 40 years). All adult patients were recruited from the Institute of Rheumatology, Prague, Czech Republic. A pediatric-onset subgroup of 66 patients was mainly selected from the Department of Pediatrics and Inherited Metabolic Disorders, Prague, Czech Republic. All patients were residents of the Czech Republic, Central-European population, with no history or signs of renal diseases and without hypolipidemic treatment. A control group of 53 normouricemic individuals was selected from personnel working at the Institute of Rheumatology (Table 1).

**Table 1:** Main demographic, biochemical, and genetic characteristics of the control (*N* = 53), asymptomatic hyperuricemic detected under 40 years of age (*N* = 72), asymptomatic hyperuricemic detected after 40 years of age (*N* = 22), intercritical gout with onset before 40 years of age (*N* = 115) and intercritical gout with onset after 40 years of age (*n* = 81) cohorts

Categorical variables	Control subjects *N* = 53		Asymptomatic hyperuricemia, detected under 40 years of age *N* = 72		Asymptomatic hyperuricemia, detected after 40 years of age *N* = 22		Intercritical gout, early onset under 40 years of age *N* = 115		Intercritical gout, onset after 40 years of age *N* = 81		
	*N*	%	*N*	%	*N*	%	*N*	%	*N*	%	**Fisher test *p*-value**
**Sex M/F**	35 / 18	66.0 / 34.0	60 / 12	83.3 / 16.7	13 / 9	59.1 / 40.9	108 / 7	93.9 / 6.1	69 / 12	85.2 / 14.8	< 0.001
**Familial occurrence**	-	-	37	56.1	3	15.0	59	55.1	22	27.8	< 0.001
**No treatment**	53	100.0	60	83.3	12	54.5	39	33.9	17	21.0	< 0.001
**Allopurinol treatment**	-	-	12	16.7	10	45.5	64	55.7	56	69.1	
**Febuxostat treatment**	-	-	0	0.0	0	0.0	12	10.4	8	9.9	
**p.Q141K**^**b**^**, MAF (Number of alleles/Allele frequency)**	5	5.7	35	24.3	6	13.6	75	32.6	37	22.8	< 0.001
**Metabolic syndrome present**^**a**^	NA	NA	2	3.2	6	27.3	13	11.8	18	23.1	< 0.001
**Continuous variables**	**Median (IQR)**	**Range**	**Median (IQR)**	**Range**	**Median (IQR)**	**Range**	**Median (IQR)**	**Range**	**Median (IQR)**	**Range**	**Kruskall-Wallis test *p*-value**
**Age of onset, years**	-	-	15.0 (7.0)	3–39	58.0 (17.5)	42–76	30.0 (12.5)	9–40	52.0 (12.2)	41–84	**-**
**Current age, years**	32.0 (16.0)	18–75	17.5 (10.5)	3–48	61.0 (20.5)	48–78	41.0 (15.0)	11–69	60.0 (14.0)	41–84	< 0.001
**BMI**	23.6 (4.0)	18.5–32	25.1 (6.7)	13.6–43.6	30.2 (3.4)	23.9–41	27.7 (5.3)	19.3–50	28.8 (5.6)	19.5–43.4	< 0.001
**CRP, mg/l**	0.8 (1.2)	0.2–8	1.1 (1.8)	0.1–153.1	3.8 (6.8)	1.1–37.9	2.7 (3.6)	0.1–268	3.8 (5.9)	0.3–224.4	< 0.001
**Serum uric acid, patients without treatment, µmol/l** **(*N* = 53 / 53 / 10 / 39 / 17) **^**c**^	338.0 (77.0)	157–420	468.0 (111.0)	361–831	451.5 (86.8)	378–631	464.0 (106.0)	181–685	469.0 (140.0)	269–683	< 0.001
**Serum uric acid, patients with treatment, µmol/l** **(*N* = 0 / 12 / 9 / 75 / 64) **^**c**^	-	-	470.0 (103.0)	328–608	407.0 (82.0)	354–487	418.0 (136.0)	217–587	453.0 (148.8)	167–647	0.499
**Serum creatinine, µmol/l**	78.0 (20.5)	51–115	74.5 (26.0)	14–140	83.0 (23.0)	54–113	81.0 (17.8)	47–213	81.0 (26.0)	48–173	0.007
**Serum cholesterol, mmol/l**	4.7 (0.9)	2.9–12.3	4.5 (1.4)	2–9.5	5.6 (1.9)	2.4–6.8	5.4 (1.4)	2.7–10.7	5.4 (1.3)	3–9.1	< 0.001
**Serum triglycerides, µmol/l**	1.0 (0.8)	0.4–3.5	1.4 (1.3)	0.6–9.1	1.8 (1.5)	0.8–5.5	1.9 (1.5)	0.6–7.5	1.8 (1.5)	0.5–6.1	< 0.001
**Serum high-density lipoproteins, µmol/l**	1.4 (0.4)	0.7–2	1.2 (0.4)	0.6–2.1	1.3 (0.4)	0.9–1.9	1.2 (0.4)	0–2.3	1.2 (0.3)	0.7–6.1	0.026
**Serum low-density lipoproteins, µmol/l**	2.8 (1.0)	1.4–5.5	3.3 (1.3)	1–6.9	3.2 (0.7)	1.9–5.1	3.4 (1.0)	0.2–9.9	3.3 (0.8)	0.7–5.7	0.001
**Atherogenic index**	2.5 (1.2)	1.4–6.4	2.6 (1.6)	1.2–6	3.2 (1.3)	1.6–4.6	3.5 (1.5)	1.2–6.8	3.5 (1.6)	1.3–7.4	< 0.001

^a^ Metabolic syndrome assessed using IDF definition; relevant data were not available for the control cohort

^b^ plus other eight rare and two novel dysfunctional variants rs372192400, rs769734146, rs200894058, rs759726272, rs140207606, rs148475733, rs199976573, rs762248204, p.T421A, and p.I242T

^**c**^ Serum uric acid measured at time of taking the analyzed sample. For 10 subjects (6 from asymptomatic hyperuricemia under 40 years group, 3 from asymptomatic hyperuricemia after 40 years group and 1 from early-onset intercritical gout group) serum uric acid measurements confirming their diagnostic status were available from different examinations only

In terms of serum UA levels, the definition of hyperuricemia was as follows: (1) men > 420 µmol/L (7.0 mg/dL) on two repeated measurements taken at least 4 weeks apart, and (2) women and children under 15 years > 360 µmol/L (6.0 mg/dL) on two repeated measurements taken at least 4 weeks apart.

The gout diagnosis was determined using criteria developed by the American College of Rheumatology (ACR) Board of Directors and the European League Against Rheumatism (EULAR) Executive Committee [26]. Patients suffering from secondary gout and other purine metabolic disorders associated with pathological concentrations of serum UA were excluded. Pediatric subjects were specifically screened for kidney disorders (uromodulin-associated disorders) and genetic metabolic disorders (glycogen storage disease, hereditary fructose intolerance, and mitochondrial disorders). Patients with these conditions were excluded from the study. The age of ascertainment (hyperuricemia) and onset (gout) was determined as the age of laboratory diagnosis in the case of asymptomatic hyperuricemia or the age of the first gout symptoms. For brevity, the term “onset” is used for both situations.

All participants/parents were fully informed about the study’s goals, and written informed consent was obtained from each participant/parent before enrollment. All tests followed standards set by the institutional ethics committees; the study was approved on 23 June 2020 as project no. 6484/2020. All procedures were performed following the Declaration of Helsinki.

### Sample preparation and lipidomic analysis

Plasma sample preparation (for lipidomic analysis) used a simple monophasic extraction with isopropanol containing internal deuterated standards (specified in supplementary table 1). The method was adopted from Sarafian et al. [27] and is described in detail in the Supplementary file. Pseudo-targeted semiquantitative lipidomic analysis, using liquid chromatography coupled to mass spectrometry (LC–MS), was adopted from Xuan et al. [28]. The LC separation was performed on an ExionLC™ System (SCIEX, Concord, CA) using reversed-phase BEH C8 column (2.1 mm, 100 mm, 1.7 µm, Waters, Milford, MA, U.S.A.), data were acquired using a QTRAP® 6500 + mass spectrometer (SCIEX, Concord, CA), and the system was controlled using Analyst software (version 1.6.2, SCIEX, Concord, CA). Complete LC–MS analysis parameters and data processing procedures are described in the supplementary file, Fig. S1, supplementary table 2, including the comparison with reference plasma material (supplementary table 3 and Fig. S2). All raw data files, supplementary tables and supplementary file were uploaded on the MassIVE database (ID MSV000093082) and are accessible under the link (https://doi.org/doi:10.25345/C54J0B76F).

### Statistical analysis

The data was processed using the R program (v 3.6.3, www.r-project.org) and the Metabol package [29]. Preprocessing included locally estimated scatterplot smoothing, Pareto scaling, filtering of analytes (with coefficients of variation in the quality control samples (QC) greater than 30%), and mean centering. Univariate statistical analysis of the data was performed and visualized using GraphPad (version 9.0, San Diego, CA, USA) and multivariate statistics with SIMCA software (version 15.0, Umetrics, Umeå, Sweden). Data were evaluated using multivariate statistical analysis (principal component analysis, PCA; orthogonal discriminant analysis by partial least squares, OPLS-DA). The univariate statistical analysis was based on the non-parametric Mann–Whitney *U* test (presented as − logs) combined with the log2 fold-change (log2 ratio of medians) and is provided in supplementary table 4. The corrected critical *p*-value for the Mann–Whitney *U* test was *p* < 8.2 × 10^−5^ (calculated from the Bonferroni correction to the number of lipids/variables corresponding to 0.05/608).

Cytoscape software (v 3.8.2, https://cytoscape.org/) was used for global visualization of the changes in lipid profiles [30]. In these lipid networks (Figs. 2,  4 and 6), each detected compound was represented by a circle (node). The connections (edges) of individual nodes were arranged according to structural similarity (lipid classes). The size of the nodes represented the − log *p*-value of the Mann–Whitney *U* test, and node color was based on fold-change (shades of red/blue represented an increase/decrease between the two groups tested corresponding to the log2 ratio of medians).

Metabolic syndrome was established using the International Diabetes Federation definition, using anamnestic data on diabetes, hypertension, and hyperlipidemia or corresponding laboratory measurements from blood samples [31]. Except for body mass index (BMI), these data were not available for the control cohort. The cohorts’ demographic, anamnestic, and laboratory characteristics were expressed as absolute and relative frequencies or medians with interquartile ranges where appropriate; they were compared using Fisher’s exact test, the Wilcoxon test, and the Kruskal–Wallis test.

## Results

### Clinical, demographic, genetic, and biochemical characterization of enrolled participants

In total, we analyzed 53 control individuals (35 male), 72 (60 male) patients with asymptomatic hyperuricemia (HUA) detected under 40 years of age (HUA ≤ 40), 22 (13 male) patients with asymptomatic HUA detected after 40 years of age (HUA > 40), 115 (108 male) patients with intercritical gout with onset under 40 years of age (Gout ≤ 40), and 81 (69 male) patients with intercritical gout with onset after 40 years of age (Gout > 40). Their demographic, anamnestic, genetic, and biochemical characteristics are presented in Table 1. Note that the HUA ≤ 40 patients had BMIs in the normal range (mean = 25.1, IQR 6.7), which was similar to the normal cohort and significantly lower than the HUA > 40 cohort (Wilcoxon test *p* < 0.001). Controls were also quite fit, with few cases of metabolic syndrome (3.2%) compared to HUA > 40 (Fisher test, *p* = 0.003). A similar observation was valid for Gout ≤ 40, where the median BMI (27.7) was significantly lower than the Gout > 40 group (*p* = 0.035); the proportion with metabolic syndrome was 11.8% (*p* = 0.035) compared to 23.1% in the Gout > 40 group). Of the HUA ≤ 40, only 12 (16.7%) patients were receiving allopurinol, compared to 10 patients (45.5%) in the HUA > 40 group. In the Gout ≤ 40, 64 (55.7%) patients were receiving allopurinol and 12 (10.4%) febuxostat compared to Gout > 40, in which 56 patients (69.1%) were taking allopurinol and 8 (9.9%) febuxostat. Other forms of ULT were not used.

Although the representation of dysfunctional variants of the ABCG2 transporter (such as p.Q141K and eight rare and two novel dysfunctional variants rs372192400, rs769734146, rs200894058, rs759726272, rs140207606, rs148475733, rs199976573, rs762248204, p.T421A, and p.I242T), previously reported [32–35] in our cohort was significantly associated with early-onset hyperuricemia and gout (*p* < 0.001), analysis of the association of dysfunctional ABCG2 variants showed none had a discernible effect on the lipid profiles of heterozygous and homozygous carriers compared to wild types based on no apparent separation of groups based on PCA and OPLS-DA (Fig. S3).

### Comparison of lipidome profiles of the hyperuricemia, gout, and normouricemic control groups

**Fig. 1:**
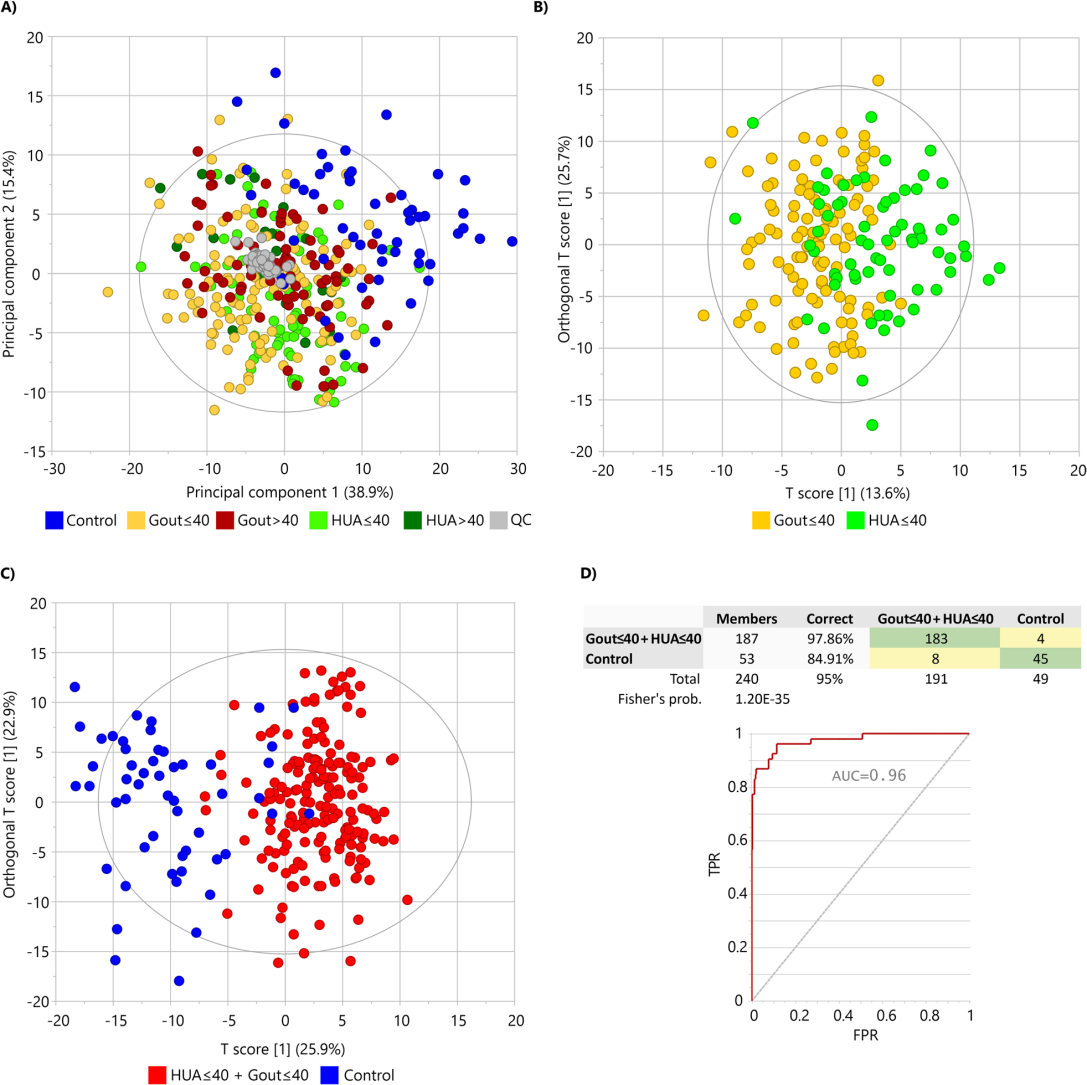
Multivariate statistical analysis indicates the differentiation of patients from controls based on plasma lipidome profile: **A** principal component analysis as an unsupervised method discriminated patient groups from healthy controls. **B** Differences between HUA ≤ 40 vs. Gout ≤ 40 patients were represented by orthogonal partial least squares—discriminant analysis (OPLS-DA). **C** OPLS-DA distinguished HUA ≤ 40 and Gout ≤ 40 patients vs. controls shown with classification performance of 95% (**D**) of the model

Unsupervised multivariate PCA provided a summary overview of the lipid profiles of all studied samples (Fig. 1A). The score plot shows the separation of the control samples from the patient samples and the clustering of patient subgroups with an overall variance explained by 54.3%. In particular, the separation of patients with early-onset or early disease detection (HUA ≤ 40 and Gout ≤ 40) was observed with PCA, indicating that their lipid profiles have been more severely impacted (Fig. 1A). As an indicator of the stability and reproducibility of the lipidomic experiment, the QC samples were closely clustered in the central region of the score plot (gray color in Fig. 1A). To evaluate differences between the HUA ≤ 40, Gout ≤ 40 patient and healthy control groups, a supervised multivariate OPLS-DA was performed. If the patient groups were compared against each other, a partial separation without statistical significance of the model (R2Y = 0.33, Q2 = 0.24) was observed (Fig. 1B) but the permutation analysis (*n* = 999) showed all R2Y values and Q2 values below the values of the original model suggesting a good fit of the model (Fig S4 A). If patient groups were statistically evaluated together against healthy controls, nearly complete separation of both two patient groups from controls was achieved (Fig. 1C) with a model classification performance of more than 95% accuracy (R2Y = 0.69, Q2 = 0.56; Fig. 1D). Permutation analysis (*n* = 999) offered Q2 < 0 and R2Y < 0.35 and all permuted models were below the original model Q2 and R2Y values (Fig. S4 B).

**Fig. 2:**
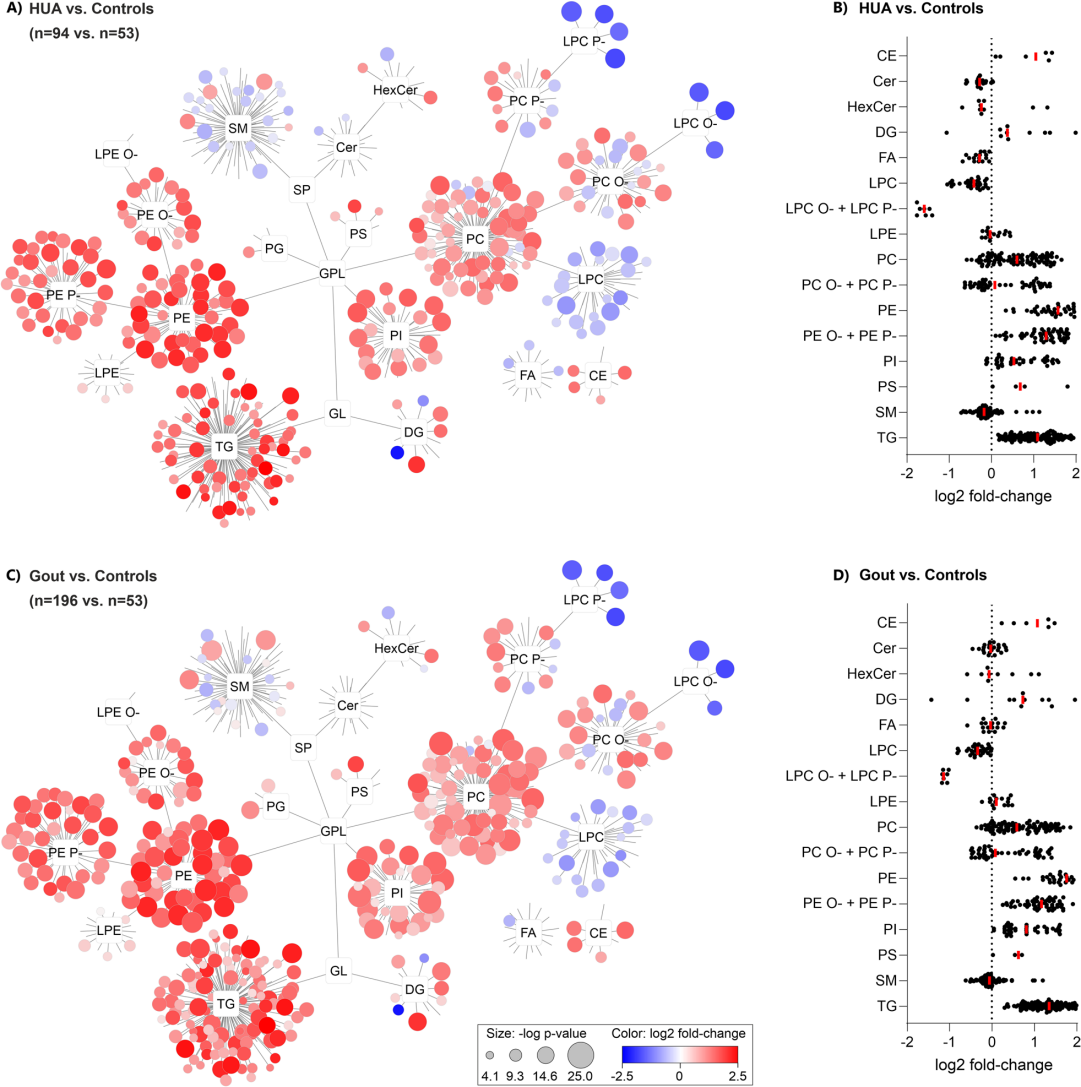
Plasma lipidome networks (**A**, **C**) and scatter plots (**B**, **D**) show similar systematic differences in the lipidome of HUA (**A**, **B**) and Gout (**C**, **D**) versus healthy controls. The most upregulated lipids were phosphatidylethanolamines (PE, median *p*-values were *p* = 2.1 × 10^−14^/*p* = 8.8 × 10^−18^ for HUA/Gout, respectively) and most downregulated were the lysophosphatidylcholine plasmalogens and plasmanyls (LPC P- and LPC O-, median *p*-values were *p* = 9.6 × 10^−18^/*p* = 3.8 × 10^−17^ for HUA/Gout, respectively). In the scatter plots (**B**, **D**), each dot represents the median value for one lipid in the corresponding class normalized by controls

For easy orientation relative to the complex lipid profile, Cytoscape networks were constructed (Figs. 2,  4, and 6) where each detected lipid species was grouped according to its class. The results show that regardless of the age of detection of hyperuricemia or gout onset, the lipidomic profiles showed similar overall trends (Fig. 2) of systematically elevated glycerophospholipids such as phosphatidylethanolamines (PE), including their plasmanyls (PE O-) and plasmalogens (PE P-), phosphatidylcholines (PC) and partially elevated plasmanyls (PC O-), plasmalogens (PC P-), phosphatidylinositols (PI), and cholesteryl esters (CE), whereas lysophosphatidylcholines (LPC) and their plasmanyls (LPC O-) and plasmalogens (LPC P-), and to some extent sphingomyelins (SM), ceramides (Cer), and free fatty acids (FA) were lower in patient groups compared to controls. The most significant upregulation was observed in PE (median fold-changes and *p*-values were 2.98/3.41 and *p* = 2.1 × 10^−14^/*p* = 8.8 × 10^−18^ for HUA and Gout versus controls, respectively) and downregulation LPC O-/P- (median fold-changes and *p*-values were 0.33/0.45 and *p* = 9.6 × 10^−18^/*p* = 3.8 × 10^−17^ for HUA and Gout versus controls, respectively). Additionally, more profound changes in the lipidome were observed in HUA ≤ 40 and Gout ≤ 40 patients compared to controls. Demonstrated, for example, by the LPC O-/P- class (median fold-changes were 0.26/0.56 and 0.35/0.56 for HUA ≤ 40/HUA > 40 and Gout ≤ 40/Gout > 40, respectively).

### Complex structural evaluation of lipidome changes in HUA and gout patients compared to controls

**Fig. 3:**
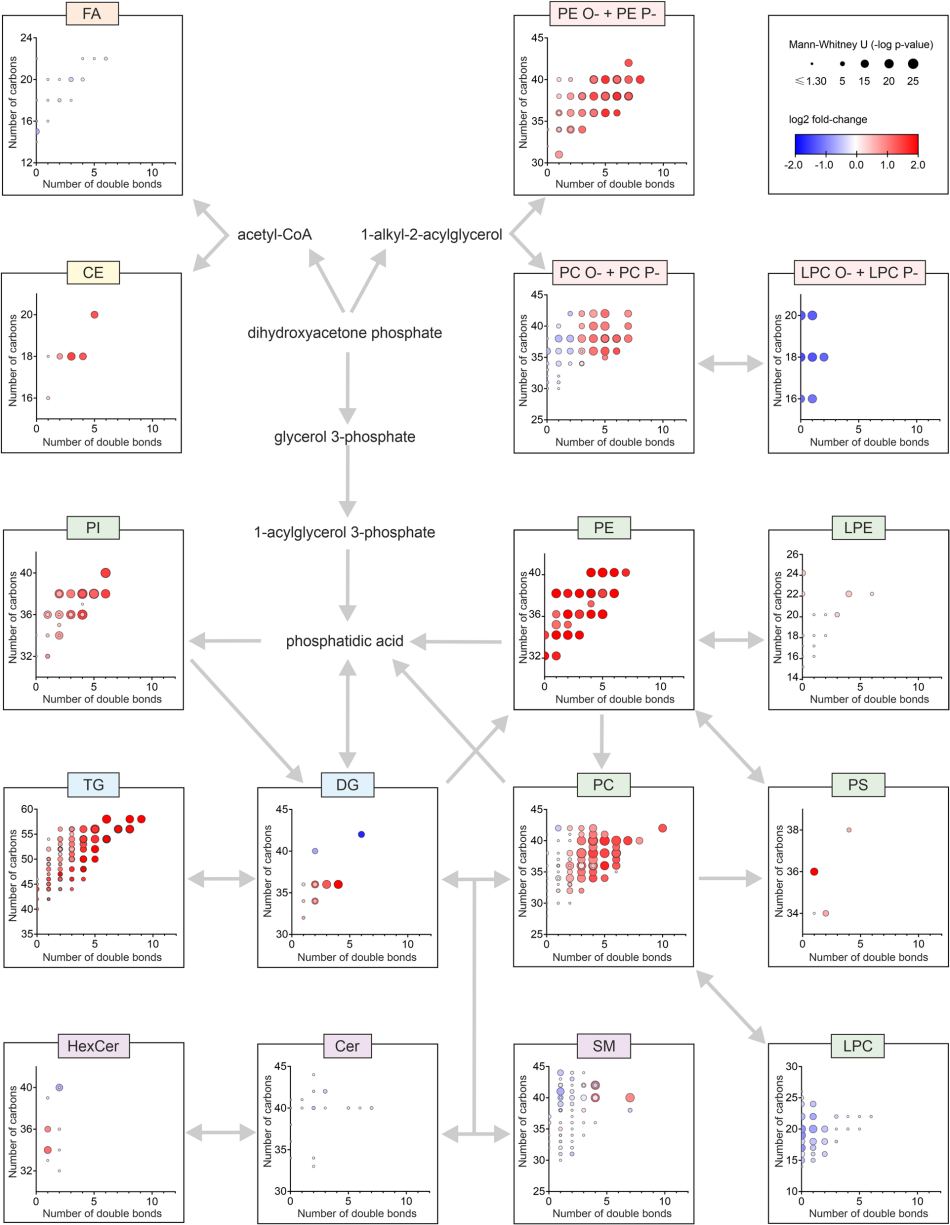
A detailed look at changes in the plasma lipidome between all patients (HUA and Gout) versus controls at the lipid structural level in the context of biochemical pathways (gray arrows) show the most statistically significant upregulations were in polyunsaturated lipids (e.g., phosphatidylcholines, PC, and their plasmalogens/plasmanyls, PC P-/O-) and triacylglycerols, TG). The *x*-axis and *y*-axis represent the number of double bonds and the number of carbons in the lipid acyl chains, respectively

To describe the pathobiochemistry of lipids in HUA and Gout patients, a detailed metabolic map of lipid chain length (number of carbons) and number of double bonds (DB) was constructed (Fig. 3). Since the observed changes in the lipidome were systematic (most of the significantly altered lipids were identical in both HUA and Gout (Fig. 2), the two groups were merged and compared against controls. Systematic lipid elevations were observed for PC O-/P-, PC, SM, LPC, and CE depending on the number of DB. Moreover, in PC O-/P-and SM, the trend was reversed from decreasing to increasing lipid levels with increasing numbers of DB. A unique situation was also found for TG, which showed the most profound changes with an increasing number of carbons and an increasing number of double bonds. The opposite was observed for LPC, where shorter and more saturated species were much lower in patients than in controls.

### Sphingolipids and glycerophospholipids accumulate in gout

**Fig. 4:**
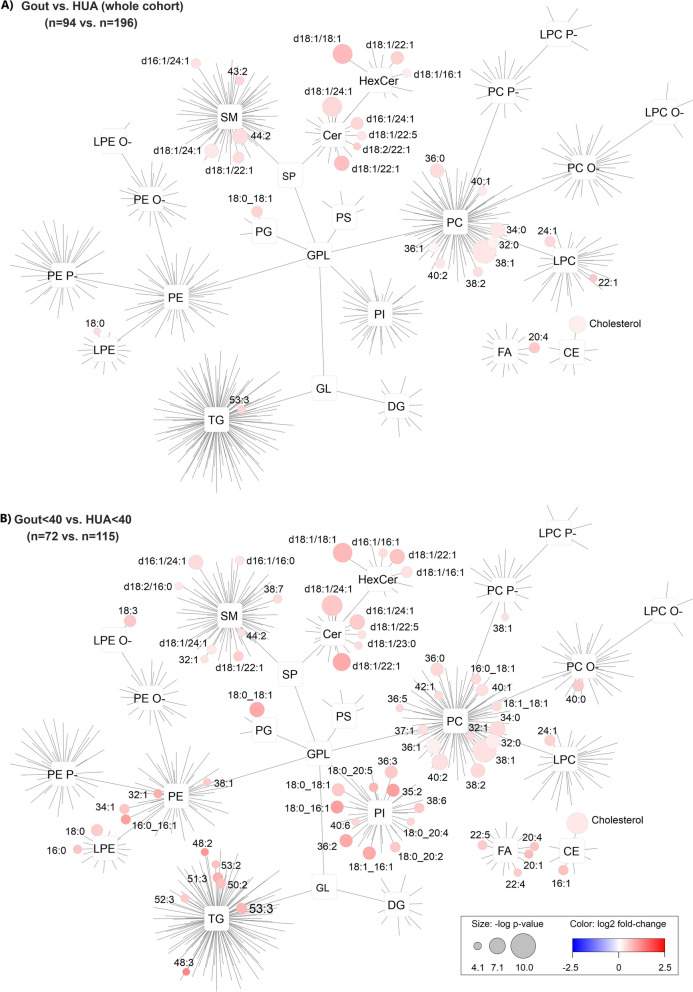
Plasma lipidome networks show significantly increased lipids in gout versus HUA for the whole cohort (**A**) and for Gout ≤ 40 versus HUA ≤ 40 groups (**B**). Sphingolipids (Cer, HexCer, and SM) and glycerophospholipids (PC, LPC, PG, and LPE) were the most significantly increased lipids in gout patients compared to HUA

After a thorough description of the significant differences in the lipidome of patients versus controls, we also focused on the differences between gout versus HUA, where, however, only a few tens of lipids were significantly altered after accounting for BF. For the entire patient cohort, significantly elevated lipids were observed from SM, Cer, HexCer, PC, PG, LPC, LPE, FA, and CE groups (Fig. 4). For example, the most significant lipids were PC 38:1, HexCer d18:1/18:1, Cer d18:1/24:1, cholesterol, SM 44:2, and Cer d18:1/22:1 with *p*-value: 2.1 × 10^−10^, 5.2 × 10^−9^, 5.4 × 10^−9^, 5.3 × 10^−8^, 1.5 × 10^−7^, and 1.8 × 10^−7^, respectively and median fold-change (gout versus HUA): 1.2, 1.6, 1.3, 1.1, 1.3, and 1.5, respectively (Fig. 4A). In the case of Gout ≤ 40 versus HUA ≤ 40, the differences were even more pronounced for some of the previously mentioned lipids PC 38:1, cholesterol, Cer d18:1/24:1, HexCer d18:1/18:1, Cer d18:1/22:1, and newly identified sphingolipids Cer d16:1/24:1 and HexCer d18:1/22:1 with *p*-value: 2.1 × 10^−10^, 1.7 × 10^−9^, 2.6 × 10^−9^, 6.6 × 10^−9^, 1.9 × 10^−8^, 1.6 × 10^−7^, and 1.9 × 10^−7^, respectively and median fold-change: 1.2, 1.2, 1.4, 1.6, 1.8, 1.4, and 1.5, respectively (Fig. 4B). Furthermore, several PE and PI were observed significantly increased in Gout ≤ 40 vs HUA ≤ 40, for example, PI 18:1_16:1, PI 18:0_16:1, and PI 18:0_18:1 with *p*-value: 8.8 × 10^−7^, 9.6 × 10^−7^, and 1.2 × 10^−6^ and median fold-change: 1.8, 1.9, and 1.4, respectively. Surprisingly, there were no significantly altered lipids found in the Gout > 40 versus HUA > 40 groups after accounting for the BF correction. This implies that lipid metabolism is more affected in gout with onset ≤ 40 years of age.

### Detailed evaluation of the effect of urate-lowering treatment on the lipid profile of patients

**Fig. 5:**
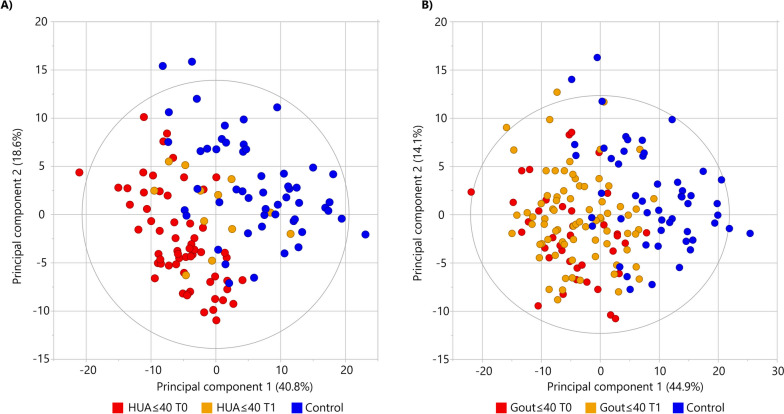
Multivariate statistics demonstrate the difference between patients and controls and the effect of urate-lowering treatment (ULT) based on the plasma lipidome. Principal component analysis of HUA ≤ 40 with ULT (T1, orange color) and without ULT (T0, red color) versus healthy controls (blue color) offers almost perfect separation (**A**) compared to Gout ≤ 40 with ULT (T1, orange color) and without ULT (T0, red color) versus healthy controls (**B**). Additionally, the HUA ≤ 40 group with ULT (T1) is shifted toward the healthy controls (**A**)

The HUA ≤ 40 and Gout ≤ 40 groups were clearly separated from the controls on PCA (Fig. 1) and showed more profound changes on the lipid network plots (details in Fig. S5). The HUA ≤ 40 and Gout ≤ 40 groups were further subdivided according to ULT (T1) or no ULT (T0). In Fig. 5A, a clear separation between the HUA ≤ 40 T0 group and controls can be observed; additionally, the HUA ≤ 40 T1 group is clustered between T0 and controls. In contrast, in Fig. 5B, the Gout ≤ 40 T0 and T1 groups overlap without any clear separation, although both groups are clearly separated from the controls.

**Fig. 6:**
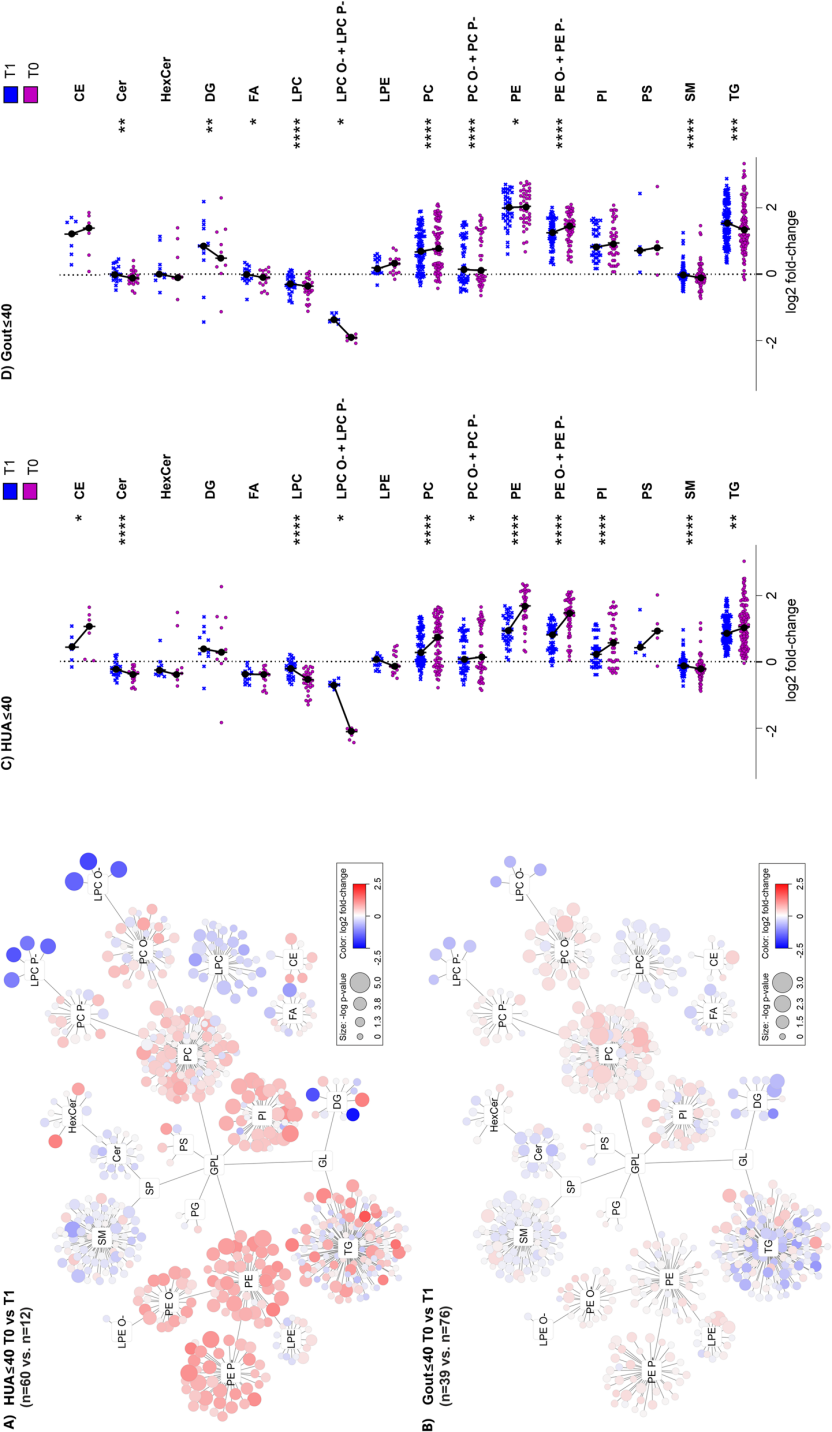
Urate-lowering treatment (ULT) shifts the lipidome profile of patients toward controls. On the plasma lipidome networks (**A**, **B**) and scatter plots (**C**, **D**), a different impact of the urate-lowering treatment on lipid profiles was observed when comparing HUA ≤ 40 and Gout ≤ 40 with or without ULT (T1/T0). Glycerophospholipids (PC, PE, PE O-, PE P-, PI, LPC O-,LPC P-) were significantly more normalized toward healthy controls in HUA ≤ 40 compared to Gout ≤ 40 (for example, in upregulated PE, the median fold-changes and original *p*-values (without Bonferroni correction) were 1.6/1.0 and 6.38 × 10^−3^/5.85 × 10^−1^ and for LPC O- and LPC P- they were 0.36/0.65 and 3.73 × 10^−4^/4.55 × 10^−^.^2^ for HUA ≤ 40 T0 vs. T1 and Gout ≤ 40 T0 vs. T1, respectively). In the scatter plots (**C**, **D**), each dot represents the median value for one lipid in the corresponding class normalized by healthy controls where the stars correspond to *p*-values (*/**/***/**** = *p* < 0.05/0.01/0.001/0.0001, respectively)

A closer look at the effect of treatment on the lipidome of patients is shown in Fig. 6. Comparing HUA ≤ 40 T0 vs. T1 in Fig. 6A, more pronounced changes can be seen relative to Gout ≤ 40 T0 vs. T1 (Fig. 6B), which is represented by the median fold-change and *p*-value (e.g., for lysophosphatidyl plasmalogens and plasmanyls, LPC O- and LPC P- as 0.36/0.65 and 3.73 × 10^−4^/4.55 × 10^−2^ and PE as 1.6/1.0 and 6.38 × 10^−3^/5.85 × 10^−1^ for HUA ≤ 40 T0 vs. T1 and Gout ≤ 40 T0 vs. T1, respectively). Due to the moderate changes in unique lipids and lower number of samples in studied groups, the Bonferroni correction (which is considered conservative and suffers from a tendency to false negatives) was not applied. Additionally, the scatter plots in Fig. 6C, D, as a more complex view, show that the median changes in lipid classes of patient groups vs. controls are more pronounced for HUA ≤ 40 T0 vs. T1 than for Gout ≤ 40 T0 vs. T1.

Glycerophospholipids (PC, PE, PE O-, PE P-, PI, LPC O-, LPC P-) were significantly more normalized toward healthy controls in HUA ≤ 40 compared to Gout ≤ 40, whereas treatment resulted in upregulation of DG and TG in Gout ≤ 40 (Fig. 6C, D).

## Discussion

The role of UA as an activator of the immune system deserves to be highlighted in the context of gout and with respect to associated comorbidities such as cardiovascular diseases and metabolic syndrome. UA is the primary antioxidant in the extracellular environment and thus can scavenge reactive oxygen species. On the other hand, UA is associated with arterial stiffness, oxidative stress, and inflammation. UA produces UA-dependent hypertension via endothelial dysfunction by reducing nitric oxide bioavailability. Elevated serum UA induces glomerular hypertension via increased vascular resistance and reduced kidney blood flow [36]. Moreover, UA is reported to be an important mediator of adipogenesis and lipogenesis. Although persistent hyperuricemia induces gout and kidney injury, the effects on metabolism and organs during the asymptomatic phase have yet to be established. Early diagnosis and implementation of required treatments are challenging without a clear understanding of the pathogenesis of hyperuricemia and gout at the molecular level.

Our study found that compared to controls, all patients had increased levels of selected unsaturated glycerophospholipids (PC, PC O-/P-, PE, PE O-/P-, and PI) and glycerolipids (TG and DG), mostly with three or more double bonds, in their plasma. In contrast, LPC and LPC O-/P- with two or fewer double bonds were decreased. Hyperuricemia is an important risk factor for gout and has significant associations with several other conditions; however, ULT of asymptomatic hyperuricemia is not routinely recommended. The effect of ULT on lipid profiles was clearly seen in HUA ≤ 40 and, to some degree, in Gout ≤ 40 patients. There was a shift toward healthy controls mainly for upregulated glycerophospholipids (PC, PE, PE O-, PE P-, PS, PI) and downregulated glycerophospholipid lysoforms (LPC, LPC O-, LPC P-). The upregulation of LPCAT3 induced by increased UA level, which has been described in the liver of hyperuricemic mice, could explain our results [25]. LPCAT3 is a phospholipid remodeling enzyme that converts LPC to PC. It prefers saturated LPC and long unsaturated acyl-CoA as substrates. Moreover, the enzyme mediates 1-O-alkyl-sn-glycerol-3-phosphocholine acylation to generate 1-O-alkyl-phosphatidylcholines [37], and it also shows activity against LPE and LPS [38]. The pathophysiological mechanisms leading to this finding are not fully elucidated but points to the involvement of LPCAT3. LPCAT3 activity is closely associated with lipid signaling proteins SREPB-1c and liver X receptor (LXR). It has been demonstrated that UA induces SREBP-1c and LXR activation. On the other hand, the Janus kinase 2/signal transducer and activator of transcription 3 (JAK2/STAT3) signaling pathway was inhibited in the liver of the mouse model with hyperuricemia [25]. JAK/STAT proteins have a role in various biological processes, for example, cell growth, apoptosis, and immune response. In lipid metabolism, loss of STAT3 or JAK2 leads to lipolysis impairment, greater body weight, and adiposity [39, 40]. SREBP-1c is a prominent regulator of lipid metabolism by activating the transcription of genes involved in lipid synthesis [41]. Cooperation between SREBP-1c and LXR has been reported to increase the synthesis of TGs in the liver and secretion into plasma as very low-density lipoproteins (VLDLs) [42]. LPCAT3 plays a critical role as it produces arachidonoyl phospholipids which are a key determinant of triglyceride secretion. Mechanistic studies indicate that arachidonoyl phospholipids are important for lipid movement within membranes and for the efficient lipidation of lipoprotein particles [43]. Mice with hepatic LPCAT3 knockdown show reduced plasma TGs, hepatosteatosis, and secretion of lipid-poor VLDL lacking arachidonoyl phospholipids [43]. Based on our data and in concordance with previous publications [25, 43], the increased LPCAT3 activity induced by increased levels of UA appears to be important in the pathobiochemistry of lipids in hyperuricemia and gout. We observed systematic trends in the saturation of lipids as well as changes directly associated with enzyme activity, expressed as decreased levels of substrates (LPC, LPC O-, and LPC P-) and increased levels of reaction products (PC, PC O-, PC P-) in patient plasma samples. Our data suggest that regulating LPCAT3 could serve as a new therapeutic target for hyperuricemia and gout in the future and should be the focus of further studies.

Furthermore, we found that changes in the lipidome of HUA ≤ 40 and Gout ≤ 40 patients were more pronounced compared to HUA > 40 and Gout > 40, even though HUA ≤ 40 and Gout ≤ 40 patients were leaner and fitter than their older counterparts. This indicates a greater impact on lipid metabolism and a greater pathobiochemical effect in younger patients with the disease. Few studies have investigated the characteristics of early-onset gout patients. One study revealed that patients with early-onset gout were more likely to have polyarticular gout flares, higher serum UA levels, and significantly higher rates of metabolic syndrome [9].

Additionally, we have focused on the changes in the plasma lipidome between gout and HUA. Generally, when we took into account the whole cohort, the most significantly increased lipids in gout patients compared to HUA belonged to the class of sphingolipids (Cer, HexCer, and SM) and a few glycerophospholipids (PC, LPC, PG, and LPE). Circulating sphingolipids have been previously associated with insulin resistance [44], cardiovascular diseases [45], obesity [46], and metabolic syndrome [47]. Cer especially have been identified as key lipotoxic players [48] as they serve as potent bioactive molecules in oxidative stress and inflammation [49], mediation of cytokines inflammatory responses [50], and apoptosis [50, 51]. Significant accumulation of sphingolipids, especially Cer, can be explained by the chronic inflammatory processes occurring in gout but not in HUA. The changes were more pronounced in Gout ≤ 40 versus HUA ≤ 40 and in addition to sphingolipids, significantly increased PI were also observed. PI containing arachidonic acid in the acyl chains are released by macrophages and regulate immune responses [52]. Additionally, the inhibition of phosphoinositide-3-kinase (PI3K), the intracellular signal transducer enzyme phosphorylating the 3-position hydroxyl group of the inositol ring of PI, induces resolution of inflammation in a mice gout model [53]. This association of PI3K with gout was further confirmed on a murine model of gout which elucidated that PI3Kγ is crucial for MSU crystal–induced acute joint inflammation and for mediating neutrophil migration and activation in gout through the regulation of caspase-1 activation [54]. However, the roles of Cer and PI in relation to chronic inflammation and immune responses in gout still have not been completely understood. Further studies should focus on more in-depth sphingolipid profiling and analysis of lipids close to the metabolism of PI such as lysophosphatidylinositols and phosphoinositides which are rarely analyzed and were also not included in our lipidomics method.

Decreased extra-renal UA excretion, caused by ABCG2 dysfunction, is a common mechanism of hyperuricemia. So far, 48 allelic variants of the *ABCG2* gene have been found, with most of the variants having significant ethnic differences in allele frequency. In contrast, only two single-nucleotide polymorphisms in the *ABCG2* gene: c.34G > A (p.V12M, rs2231137) and c.421C > A (p.Q141K, rs2231142), are recognized as common variants across most ethnic groups [55]. The p.Q141K variant impacts the age of hyperuricemia and gout onset (earlier disease onset *p* = 0.004) and the trend toward lower BMIs (*p* = 0.056) and lower levels of C-reactive protein (*p* = 0.007); it also leads to higher glomerular filtration rates (*p* = 0.035) [56]. Several studies have reported that ABCG2 is essential in stimulating inflammation and regulating autophagy. However, in our study, we did not find a relationship between p.Q141K carriers and changes in lipid profiles; on the other hand, we found significant changes in the early-onset group.

In a previously published cross-sectional observational study, patients with early-onset gout had more frequent gout attacks and more clinically affected joints but fewer cardiovascular, cerebrovascular, and renal comorbidities at disease presentation; however, they were nonetheless at increased risk of cardiovascular events [10]. Moreover, a stratified analysis conducted as a part of a meta-analysis of cohort studies found a gradient of increased risk of myocardial infarction relative to younger ages at gout onset [57]. The mechanisms of gout may differ between early-onset and late-onset since the clinical features of these two groups are different. The main metabolic differences involve lipid metabolism (LPC class as the main part of oxidized LDL). High concentrations of LPCs were found within atherosclerotic plaques, suggesting the role of LPCs in developing endothelial dysfunction and atherosclerosis [58, 59]. In another study, early-onset juvenile gout patients had significantly higher BMI, serum UA level, total cholesterol, triglyceride, LDL-C, glutamate oxaloacetate transaminase, glutamate pyruvate transaminase, and serum creatinine, and significantly lower HDL-C and lower glomerular filtration rates [60].

Our study has several strengths. Patient cohorts and controls were collected at a single tertiary center in the Czech Republic. All steps involving subject selection, sample collection and storage, and lipidomic analysis were strictly controlled to ensure the most reproducible results. A sensitive targeted lipidomic analysis with high coverage (over 600 lipids in plasma) and structural identification (acyl-specific analysis: length and number double bonds) was performed on 343 samples (290 patients and 53 controls), which is unique compared to previous studies. Liu et al. [22] analyzed 428 serum samples (340 patients and 88 controls) but only provided information on 245 identified metabolites/lipids. In another study, Liu et al. [25] identified 812 lipids but only analyzed 200 serum samples (100 patients and 100 controls).

Study limitations include imbalances in the sex and age of the control and patient groups. This was due to differences in the prevalence of hyperuricemia and gout between men and women. However, both factors were considered, and no significant effects on study outcomes were observed (Fig. S6 and Fig. S7). All cohort patients and controls were Caucasian. Information regarding alcohol intake was not collected. Additionally, our instrumentation was not capable of complete structural identification of lipids with respect to the position and stereochemistry of double bonds (which, on the other hand, is usually less sensitive and robust) and did not include every lipid class that can be observed in plasma (due to the limitation of simultaneous analysis of a large number of lipids).

## Conclusions

Gout and hyperuricemia are associated with alterations in plasma lipid profiles (namely increased glycerophospholipids and TG and decreased LPC, LPC O-, and LPC P-). These changes are significantly more pronounced in early-onset or early disease detection. This change cannot be explained based on the genetics of ABCG2 or by common features of metabolic syndrome, such as BMI. In our study, ULT had a significant effect on the normalization of lipid profiles, especially on HUA ≤ 40 patients, while in groups > 40 years, this trend was not evident. The benefits of early initiation of ULT in early-onset hyperuricemia patients require careful analysis. Additionally, more discussion of using a personalized approach for managing hyperuricemia in clinical practice is necessary.

The different lipid concentrations and altered biochemical pathways are likely due to increased UA in HUA and gout, highlighting the pathobiochemical mechanism at the molecular level of lipids in hyperuricemia progressing to gout. Furthermore, we found that in gout compared to HUA there is an accumulation of sphingolipids (mainly Cer) and in Gout ≤ 40 additionally PI, which could be associated with pathophysiological processes of chronic inflammation and immune response. Additional studies are needed to confirm potential biomarkers and new targets for the treatment of gouty arthritis.

### Supplementary Information

**Additional file 1: Supplementary File.** Detailed characterization of the sample preparation, LC-MS lipidomic analysis, data processing and semiquantification of lipids. **Fig. S1.** Lipid patterns plotted separately for each lipid class. **Fig. S2.** Accuracy assessment for SRM 1950 - "Metabolites in Frozen Human Plasma" (number of independently prepared replicates: n=10). **Fig. S3.** PCA (A) and OPLS-DA (B) analysis showing the effect of the dysfunctional mutation of the ABCG2 gene (such as p.Q141K and other mutations with the same dysfunctional effect) on the lipidome of all patients compared by wildtype (WT), heterozygous (HET) or homozygous (HOM) gene inheritance. **Fig. S4.** Validation of the OPLS-DA models from Figure 1 B (A) and Figure 1 C (B) based on permutation test performed with 999 permutations. **Fig. S5.** Overview of lipid networks based on hyperuricemia (HUA), gout and age of onset/detection ≤/>40 years and urate-lowering therapy (T0/T1) versus controls. **Fig. S6.** Influence of sex on the changes in lipidome comparing hyperuricemia (HUA) and gout versus controls. **Fig. S7.** Influence of age on the changes in lipidome comparing hyperuricemia (HUA) and gout versus all controls and age-matched controls.

**Additional file 2: Supplementary Table 1. **Specifications of used internal standards.

**Additional file 3: Supplementary Table 2. **Semiquantitative concentrations (nmol/mL) of all measured lipids in all samples including LC-MS parameters for measured lipids.

**Additional file 4: Supplementary Table 3. **Comparison of semiquantitative concentrations (nmol/mL) in SRM NIST 1950 (n=10) with reference values (LipidQC export).

**Additional file 5: Supplementary Table 4. **Statistical evaluation of the data by log2 fold change and -log p-value of Mann-Whitnney U test.

## Data Availability

The datasets generated and/or analyzed during the current study are available in the MassIVE repository (ID MSV000093082); (https://doi.org/doi:10.25345/C54J0B76F).

## Abbreviations

ABCG2: ATP-binding cassette subfamily G member 2
ACN: Acetonitrile
ACR: American College of Rheumatology
BMI: Body mass index
CE: Cholesteryl esters
Cer: Ceramides
DB: Double bonds
DG: Diacylglycerols
EULAR: European League Against Rheumatism
FA: Free fatty acids
Gout > 40: Patients with intercritical gout with onset after 40 years of age
Gout ≤ 40: Patients with intercritical gout with onset under 40 years of age
HDL-C: High-density lipoprotein cholesterol
HexCer: Hexosylceramides
HUA: Hyperuricemia
HUA > 40: Patients with asymptomatic hyperuricemia detected after 40 years of age
HUA ≤ 40: Patients with asymptomatic hyperuricemia detected under 40 years of age
IPA: Isopropanol
JAK2/STAT3: Janus kinase 2/signal transducer and activator of transcription 3
LC-MS: Liquid chromatography coupled to mass spectrometry
LDL-C: Low-density lipoprotein cholesterol
LPC O-: Lysophosphatidylcholine plasmanyls
LPC P-: Lysophosphatidylcholine plasmalogens
LPC: Lysophosphatidylcholines
LPCAT3: Lysophosphatidylcholine acyltransferase 3
LPE O-: Lysophosphatidylethanolamine plasmanyls
LPE: Lysophosphatidylethanolamines
LXR: Liver X receptor
MSU: Monosodium urate
OPLS-DA: Orthogonal discriminant analysis by partial least squares
PC O-: Phosphatidylcholine plasmanyls
PC P-: Phosphatidylcholine plasmalogens
PC: Phosphatidylcholines
PCA: Principal component analysis
PE O-: Phosphatidylethanolamine plasmanyls
PE P-: Phosphatidylethanolamine plasmalogens
PE: Phosphatidylethanolamines
PG: Phosphatidylglycerols
PI: Phosphatidylinositols
PI3K: Phosphoinositide 3 kinase
PS: Phosphatidylserines
QC: Quality control samples
SLC2A9: Solute carrier family 2 member 9
SM: Sphingomyelins
SREBP-1c: Sterol regulatory element-binding protein-1c
TG: Triacylglycerols
UA: Serum uric acid
ULT: Urate-lowering therapy/treatment
VLDLs: Very low-density lipoproteins
