# Supplementary material for: Alterations in lipidome profiles distinguish early-onset hyperuricemia, gout, and the effect of urate-lowering treatment
Source: Arthritis Res Ther. 2023 Dec 2;25:234. doi: 10.1186/s13075-023-03204-6 (PMC10693150; doi:10.1186/s13075-023-03204-6)

**Alterations in Lipidome Profiles Distinguish Early-Onset Hyperuricemia, Gout, and the Effect of Urate-Lowering Treatment**

Aleš Kvasnička^1^, David Friedecký^1^, Radana Brumarová^1^, Markéta Pavlíková^2^, Kateřina Pavelcová^3^, Jana Mašínová^3^, Lenka Hasíková^3^, Jakub Závada^3^, Karel Pavelka^3^, Pavel Ješina^4^, Blanka Stibůrková^3,4*^

^1^Laboratory for Inherited Metabolic Disorders, Department of Clinical Biochemistry, University Hospital Olomouc and Faculty of Medicine and Dentistry, Palacký University Olomouc, Czech Republic

^2^Department of Probability and Mathematical Statistics, Faculty of Mathematics and Physics, Charles University, Prague, Czech Republic

^3^Institute of Rheumatology, Prague, Czech Republic

^4^Department of Pediatrics and Inherited Metabolic Disorders, Charles University-First Faculty of Medicine and General University Hospital in Prague, Prague, Czech Republic

*Corresponding author

Address of correspondence:
Blanka Stibůrková
Institute of Rheumatology, Na Slupi 4, 128 50 Praha 2, Czech Republic
ORCID: [0000-0003-2465-1476](https://orcid.org/0000-0003-2465-1476)
Email: stiburkova@revma.cz

**Sample preparation, LC-MS lipidomic analysis, data processing and semiquantification of lipids**

**Sample preparation**. Plasma samples were collected and stored at -80 °C until analysis. Samples were firstly brought from ‑80 °C to -20 °C overnight and the next day they were freeze-thawed on ice. Directly after freeze-thawing and 10 s mixing on a vortex mixer, the extraction was carried out by mixing 50 µl of plasma with 150 μl of IPA containing internal standards (specified in Supplementary Table 1) and vortexing for 10 s as described in Sarafian *et al.* (1). Samples were left overnight in the freezer (-20 °C) for deproteination. The next day the mixture was centrifuged (10 min, 14 000 g, 4 °C) and the supernatant above the protein pellet was pipetted (approximately 150 µl) into a glass LC-MS vial. An aliquot of 10 µl from each sample was collected for the quality control (QC) sample. The samples were then immediately subjected to liquid chromatography coupled to mass spectrometry (LC-MS) analysis. Double randomization of samples was carried out, first during the sample preparation and second regarding the analytical sequence. QC sample was measured as every 6th injection and was used for the instrument stability monitoring.

**LC-MS lipidomic analysis**. Lipids were semiquantified using a validated pseudotargeted lipidomic method adopted from Xuan *et al.* (2). The LC separation was performed on an ExionLC™ System (SCIEX, Concord, CA), data were acquired using a QTRAP® 6500+ mass spectrometer (SCIEX, Concord, CA), and the system was controlled using Analyst software (version 1.6.2, SCIEX, Concord, CA). A reversed-phase BEH C8 column (2.1 mm, 100 mm, 1.7 µm, Waters, Milford, MA, U.S.A.) was used for the chromatographic separation. Mobile phase A consisted of ACN:H_2_O (3:2, v/v), the mobile phase B was IPA:ACN (9:1, v/v), and both contained 10 mM ammonium acetate. The flow rate was set at 0.35 ml/min and the column was tempered at 55 °C. The elution gradient started with 32% B up to 1.5 min, then linearly increased to 85% B at 15.5 min, then it increased again to 97% B at 15.6 min, was kept for 2.4 min. The gradient then reached its initial composition of 32% B at 18.1 min and it was kept for 1.9 min for column equilibration. The parameters of the ion source and gasses of the mass spectrometer were set as follows: ion spray voltage, +4500 V and -4500 V; curtain gas, 40 psi; both ion source gasses 1 and 2, 60 and 50 psi respectively, and source temperature, 400 °C. Scheduled multiple reaction monitoring with a window of 2 minutes was applied for the data acquirement. Positive and negative ionization of compounds in one analysis was performed, using the polarity-switching ability of the used mass analyzer. Specific acyl-defining MRM transitions were calculated using LipidCreator software (3) and they were added to the method for the identification of lipid molecular species (acyl-specific identification). Declustering potentials and collision energies for each lipid class were optimized with the SPLASH Lipidomix Mass Spec standard (Avanti Polar Lipids, Alabaster, AL) and same as the linearity and other analytical parameters they have been already provided in detail in our previous work utilizing the same lipidomics approach (4). Lipid pattern elution curves plotted via the R script in the R program (version 3.6.3.) created by Drotleff *et al.* (5) were used to verify correct lipid annotation (Supplementary Figure 1).

**Data processing.** Data from lipidomic analysis were processed in SCIEX OS software (Sciex, version 1.6.1). The peak areas corresponding to each lipid were divided by the areas of their internal standards (always one internal standard for one or more lipid classes or subclasses was used, provided in detail in Supplementary Table 1). The data preprocessing and statistical analysis using the R program is described in the main article text. Based on Hotelling’s T2Range (according to the OPLS-DA model) and visual inspection of the PCA, one outlier control sample was removed from the final dataset. All raw data files, Supplementary Tables and Supplementary File were uploaded on the MassIVE database (ID MSV000091596) and are accessible under the link (https://doi.org/doi:10.25345/C5639KF91).

**Semiquantification of lipids**. In contrast to the original method, where odd-chained internal standards were used, our approach used a deuterated mix of standards (SPLASH® LIPIDOMIX® Mass Spec Standard mixture, Avanti Polar Lipids, Alabaster, AL, USA) with the addition of labeled ceramide (Cer d18:1-d7/15:0) and oleic acid-d9 (FA 18:0-d9) more information provided in the Supplementary Table 1. One labeled standard was always used to semiquantify one or more corresponding lipid classes or subclasses (shown in detail in the Supplementary Table 1). As reversed phase liquid chromatography separation was used and only one standard per lipid (sub)class was utilized, the same ionisation conditions (due to matrix effects) could not be guaranteed resulting in a not a fully quantitative data but rather only semiquantitative (level 3). This problem is well known in lipidomics, but a recent study showed that many lipid classes can also be sufficiently quantified while using reverse phase separation utilizing the “one ISTD-per-lipid class” approach (6). For this reason, although we report lipid concentrations in nmol/mL, we claim that these are semiquantitative data. In the case of lipids defined by both acyl chains (not by a summed name), the concentration values in nmol/mL are also semiquantitative as multiple standards (correcting for different acyl chain lengths and different numbers of double bonds) were not used, therefore the same quantification fragment was not used, and response factors were not applied. This applies to the analysis of PC, PE, PI, PG and PS in the negative mode by MRM by transition to their acyl chain and to the analysis of SM long chain base (LCB) in positive mode. Concentrations calculated in this way (for which a type I correction was applied) corresponds to the Lipidomics Standards Initiative guidelines (7) and is considered as semiquantitative (level 3, Supplementary Table 2). Although lipids were only semiquantitatively determined by this analytical approach, a comparison of the concentration values in the standard reference material (SRM NIST 1950, number of independently prepared replicates: n = 10) with the reference values is provided (Supplementary Figure 2 and Supplementary Table 3). This comparison was made using a Microsoft Excel application LipidQC (8) based on the National Institute of Standards and Technology (NIST) Lipidomic Interlaboratory Comparison Exercise (NIST-ILCE). LipidQC only includes reference comparisons for lipid species measured by five or more participating laboratories with coefficient of dispersion (COD) values of ≤40%. Results from LipidQC are provided below in the Supplementary Figure 2 and Supplementary Table 3.

**References**

1. Sarafian MH, Gaudin M, Lewis MR, Martin FP, Holmes E, Nicholson JK, Dumas ME. Objective set of criteria for optimization of sample preparation procedures for ultra-high throughput untargeted blood plasma lipid profiling by ultra performance liquid chromatography-mass spectrometry. Anal Chem. 2014 Jun 17;86(12):5766-74. doi: 10.1021/ac500317c. Epub 2014 Jun 2. PMID: 24820162.

2. Xuan Q, Hu C, Yu D, Wang L, Zhou Y, Zhao X, Li Q, Hou X, Xu G. Development of a High Coverage Pseudotargeted Lipidomics Method Based on Ultra-High Performance Liquid Chromatography–Mass Spectrometry. Analytical Chemistry. 2018;90(12):7608–7616.

3. Peng B, Kopczynski D, Pratt BS, Ejsing CS, Burla B, Hermansson M, Benke PI, Tan SH, Chan MY, Torta F, Schwudke D, Meckelmann SW, Coman C, Schmitz OJ, MacLean B, Manke MC, Borst O, Wenk MR, Hoffmann N, Ahrends R. LipidCreator workbench to probe the lipidomic landscape. Nat Commun. 2020 Apr 28;11(1):2057. doi: 10.1038/s41467-020-15960-z. PMID: 32345972; PMCID: PMC7188904.

4. Kvasnička A, Friedecký D, Tichá A, Hyšpler R, Janečková H, Brumarová R, Najdekr L, Zadák Z. SLIDE-Novel Approach to Apocrine Sweat Sampling for Lipid Profiling in Healthy Individuals. Int J Mol Sci. 2021 Jul 28;22(15):8054. doi: 10.3390/ijms22158054. PMID: 34360820; PMCID: PMC8348598.

5. Drotleff B, Roth SR, Henkel K, Calderón C, Schlotterbeck J, Neukamm MA, Lämmerhofer M. Lipidomic profiling of non-mineralized dental plaque and biofilm by untargeted UHPLC-QTOF-MS/MS and SWATH acquisition. Anal Bioanal Chem. 2020 Apr;412(10):2303-2314. doi: 10.1007/s00216-019-02364-2. Epub 2020 Jan 15. PMID: 31942654; PMCID: PMC7118048.

6. Lange M, Fedorova M. Evaluation of lipid quantification accuracy using HILIC and RPLC MS on the example of NIST® SRM® 1950 metabolites in human plasma. Analytical and Bioanalytical Chemistry. 2020;412(15):3573–3584.

7. Lipidomics Standards Initiative Consortium. Lipidomics needs more standardization. Nat Metab. 2019 Aug;1(8):745-747. doi: 10.1038/s42255-019-0094-z. PMID: 32694765.

8. Ulmer CZ, Ragland JM, Koelmel JP, Heckert A, Jones CM, Garrett TJ, Yost RA, Bowden JA. LipidQC: Method Validation Tool for Visual Comparison to SRM 1950 Using NIST Interlaboratory Comparison Exercise Lipid Consensus Mean Estimate Values. Analytical Chemistry. 2017;89(24):13069–13073.

**Fig. S1** Lipid patterns plotted separately for each lipid class. Charts are plotted as *m/z* value on the y-axis and retention time in minutes on the x-axis. Saturation equals to the number of double bonds. Lipid pattern plots were used to correctly assign lipid annotations and to annulate potential misidentifications. Few shifts can be observed for example in Cer and HexCer with 1 double bond, which is occurring due to different long-chain base composition (d16:1, d18:1, or d20:1). Lipid isomers with different acyl-composition can also differ in retention time resulting in non-absolute linear/quadratic pattern curve observed (for example in the case of PC 36:4 where both PC 18:2_18:2 and PC 20:4_16:0 species were observed). Additionally, patterns are provided for PC and PE species measured in negative mode (PCFA, PEFA).


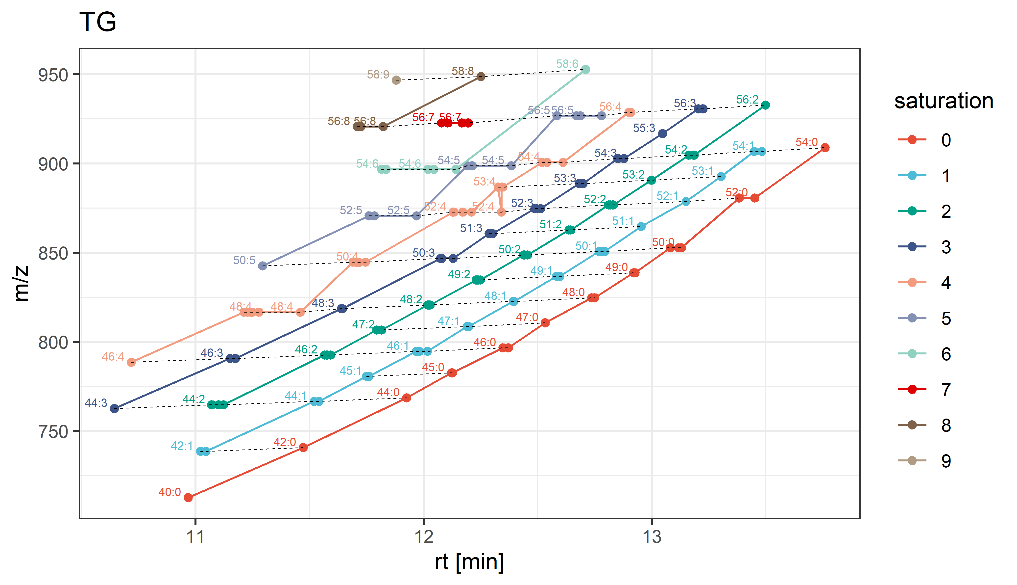

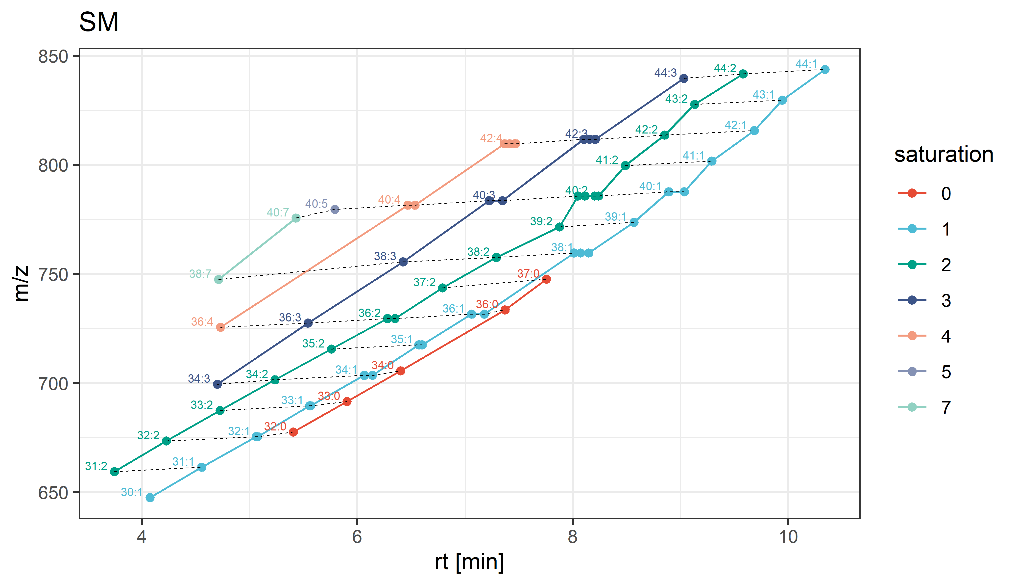

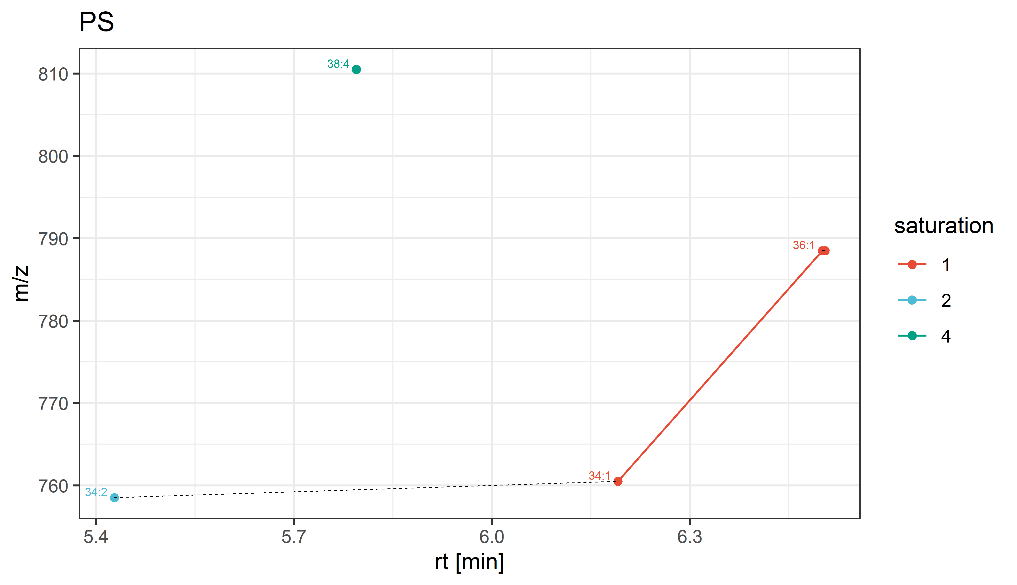

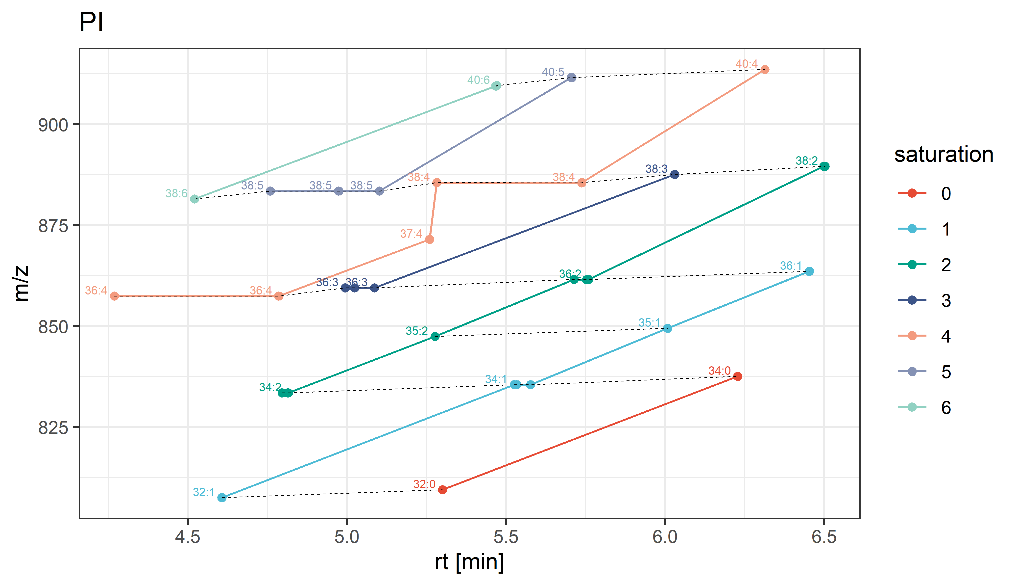

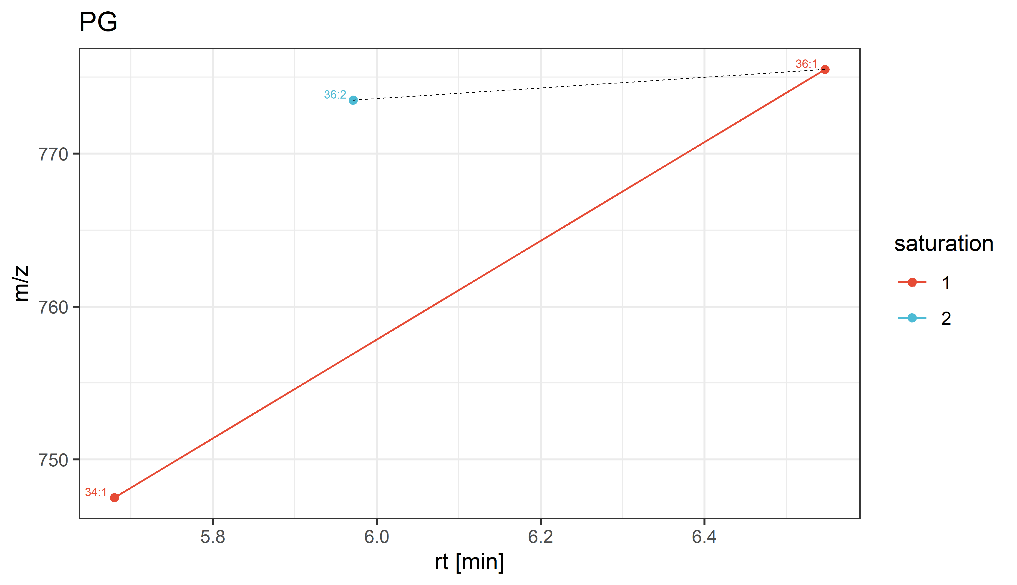

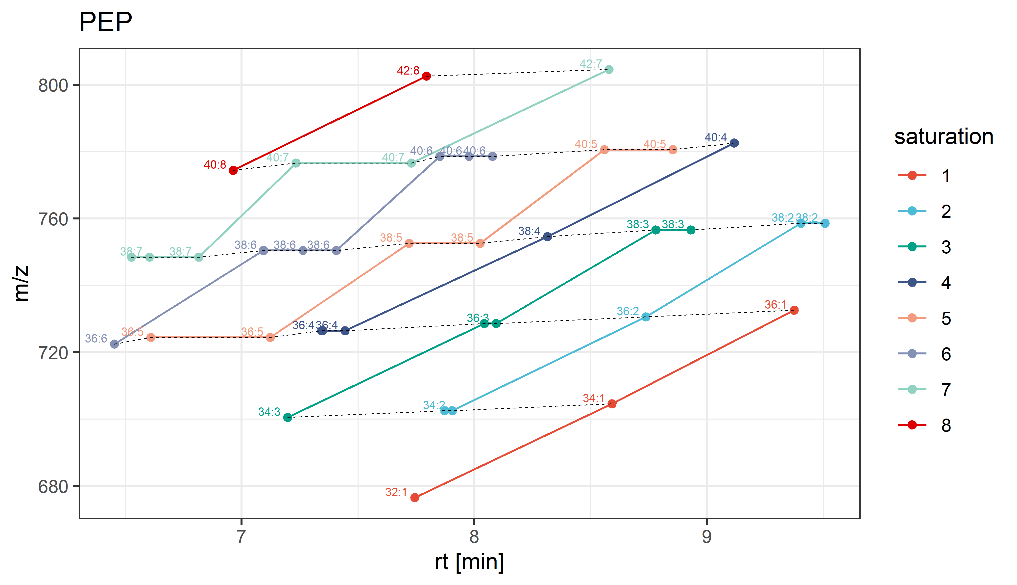

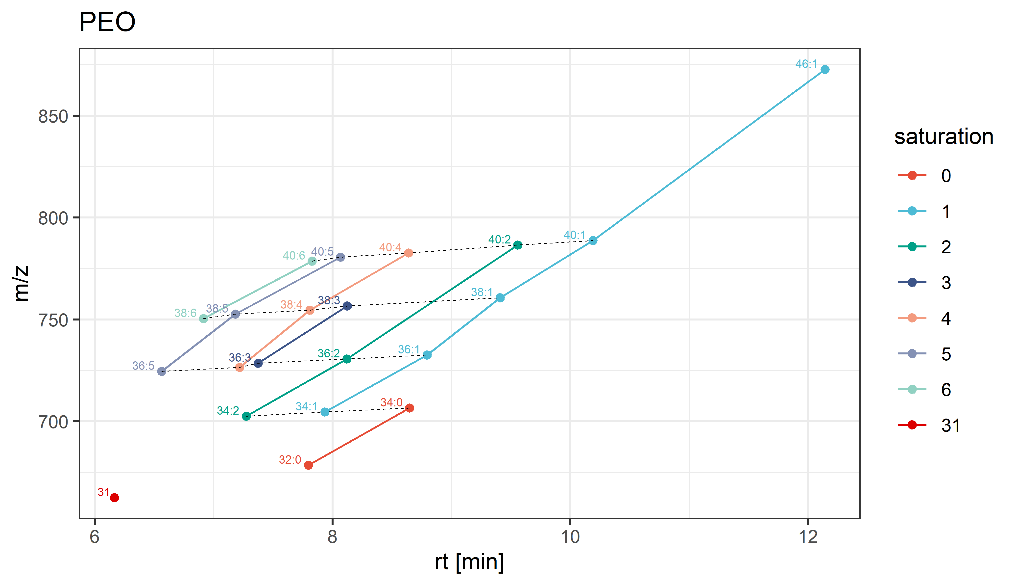

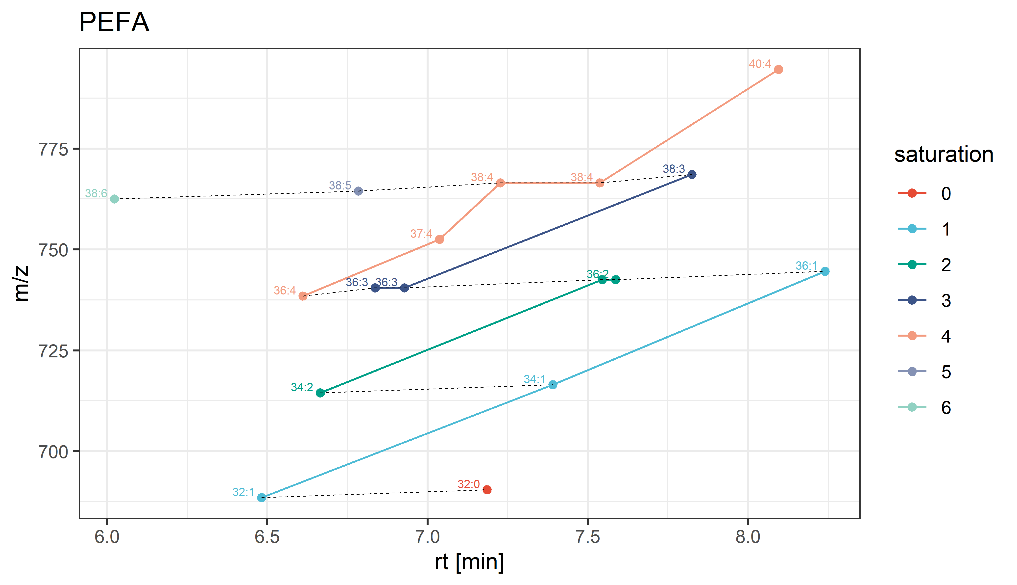

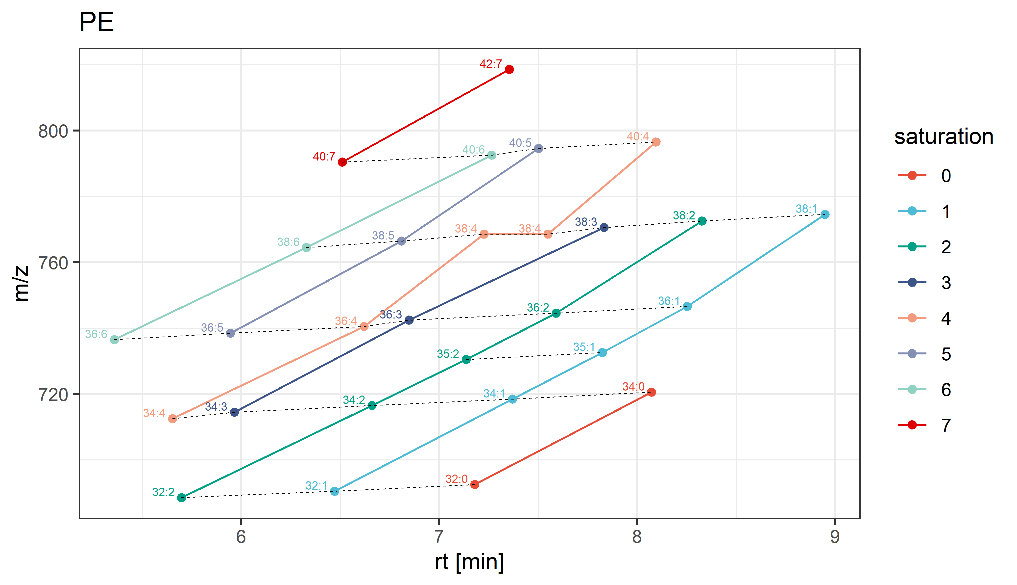

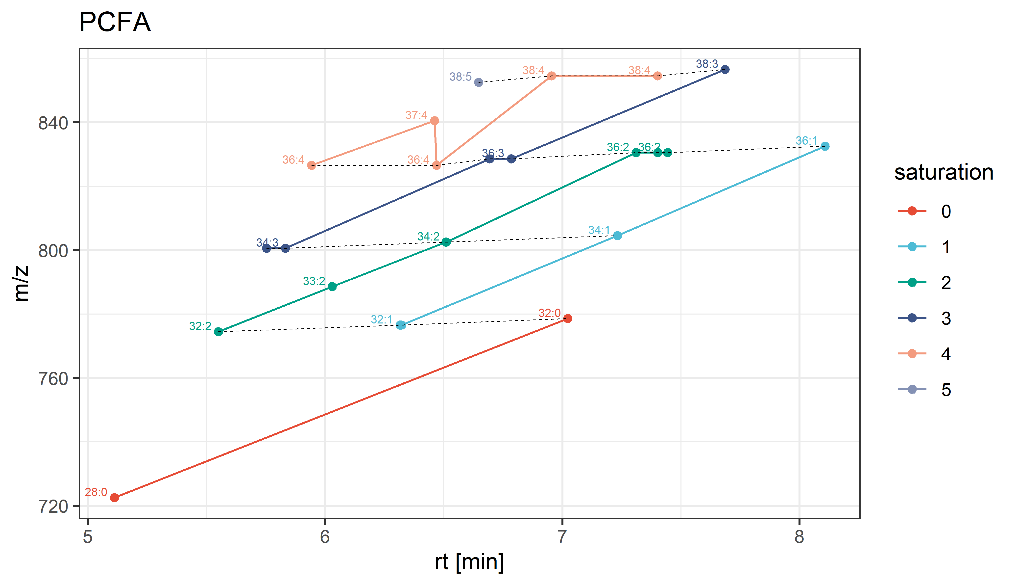

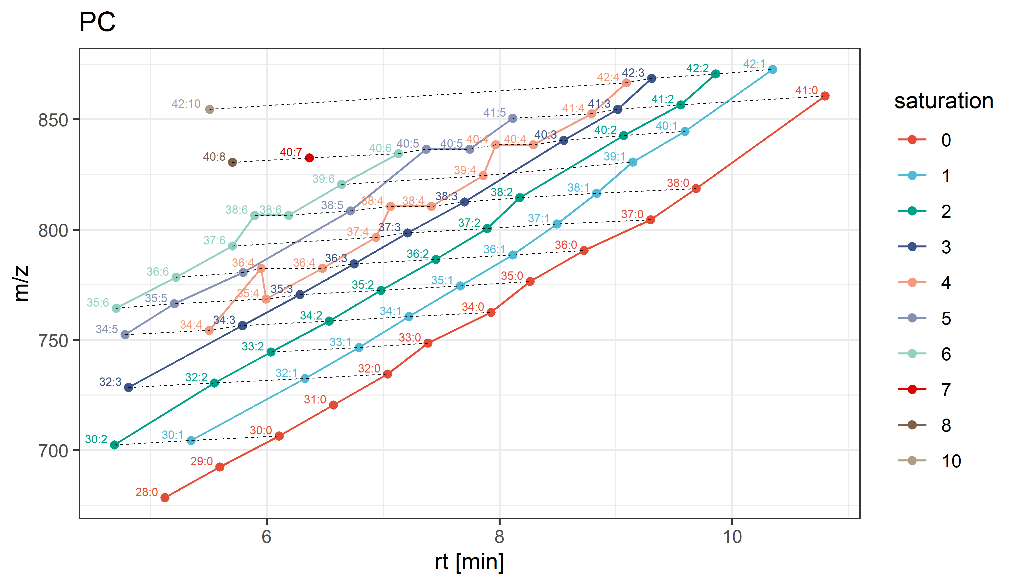

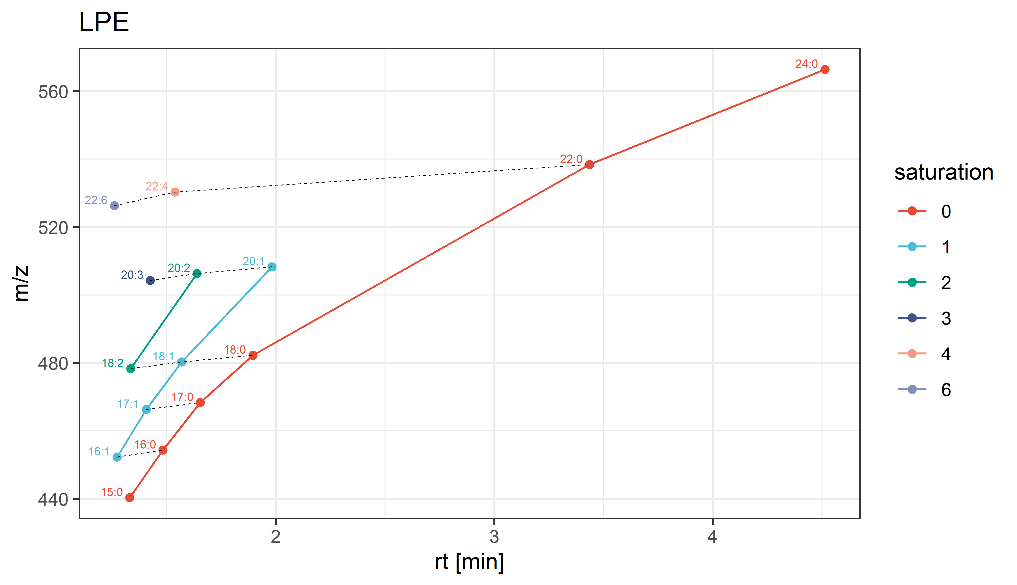

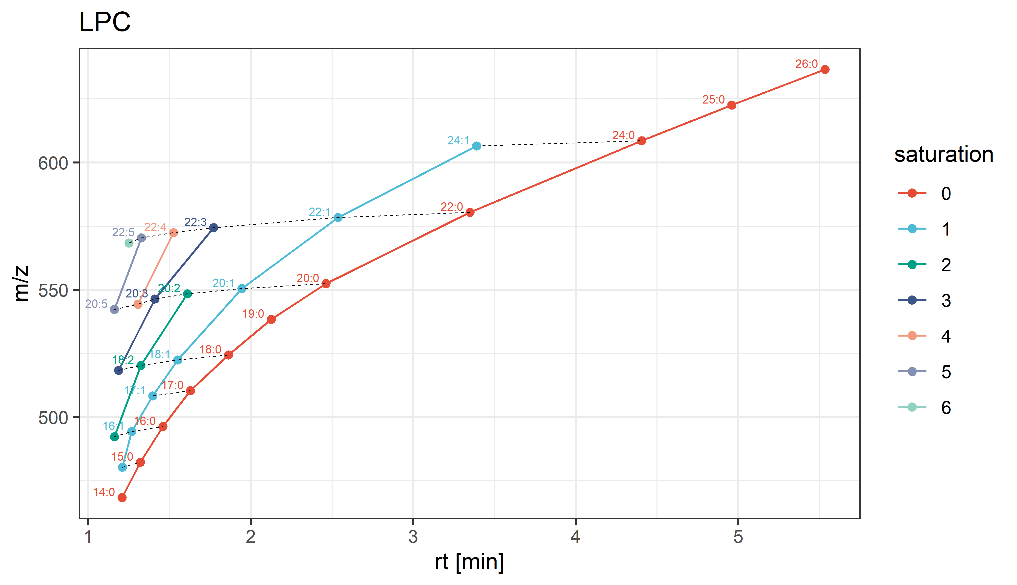

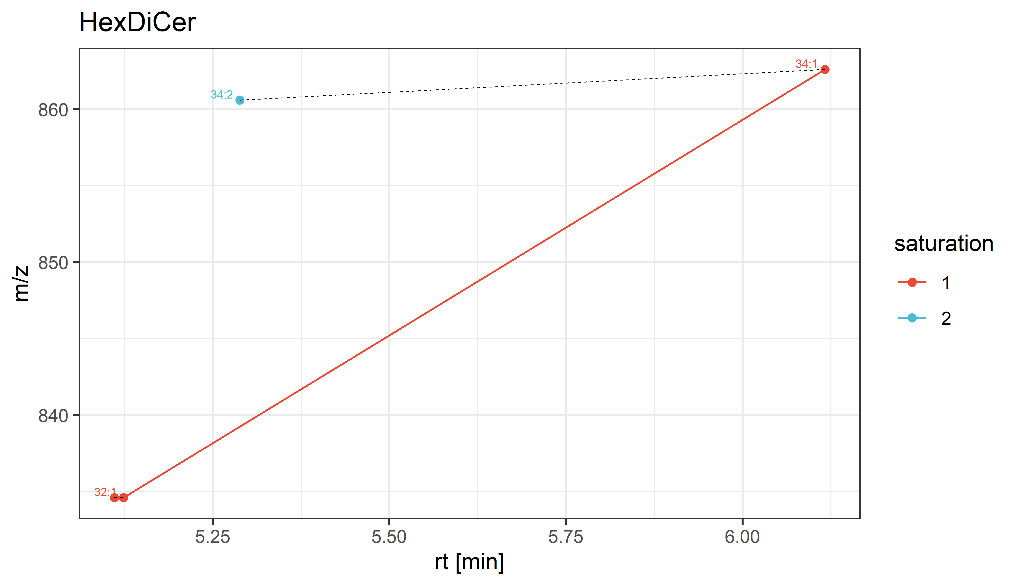

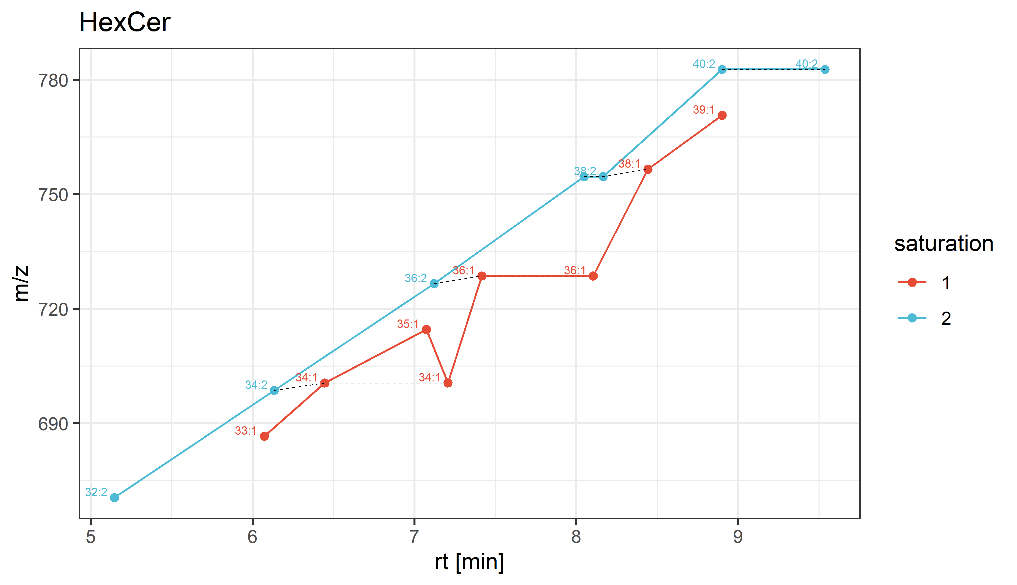

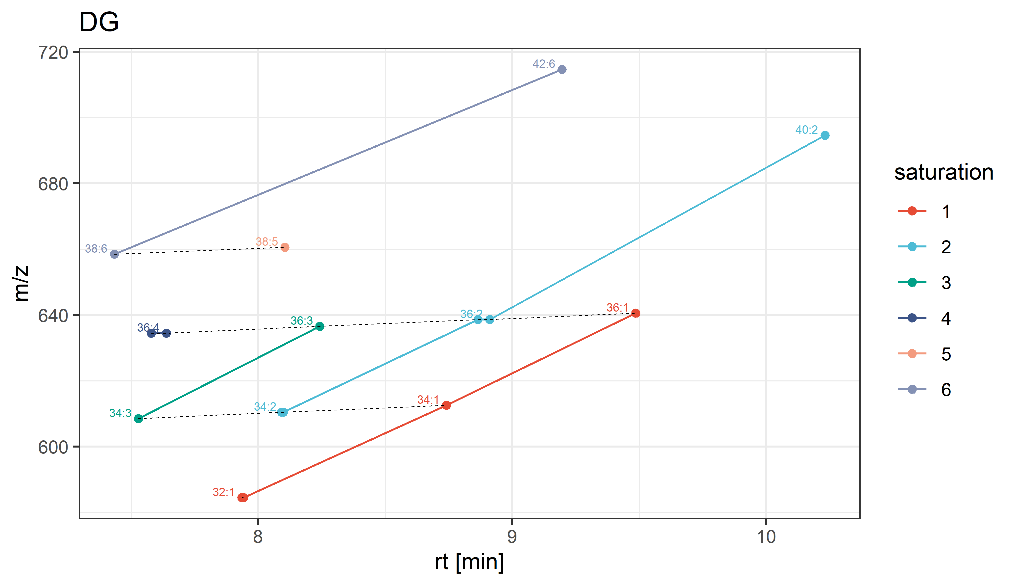

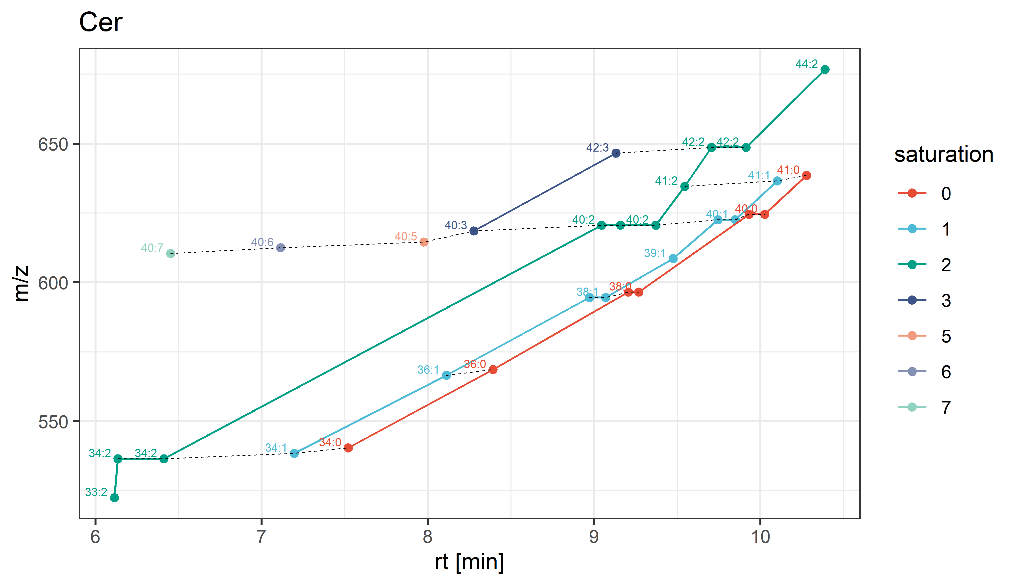

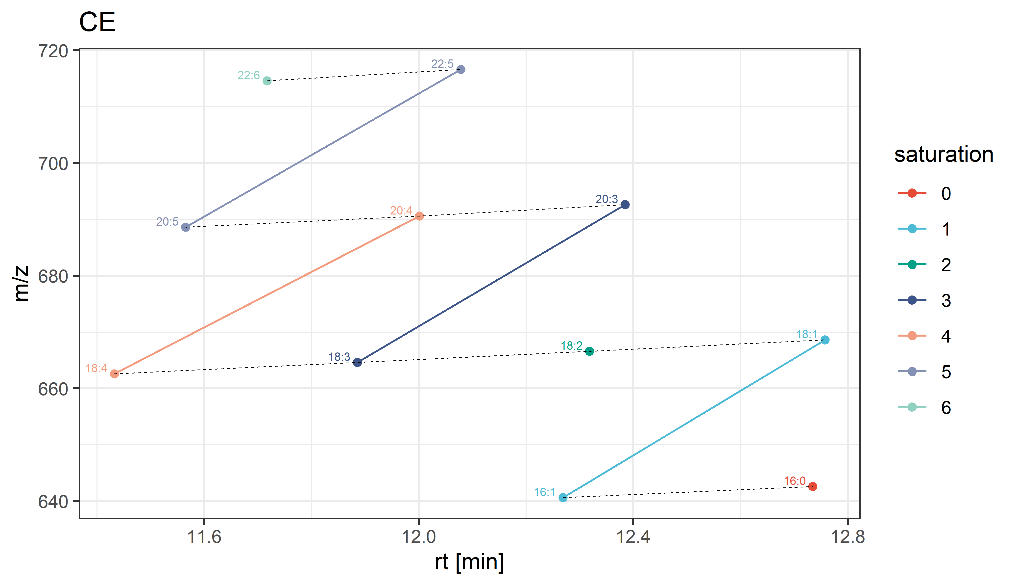
**
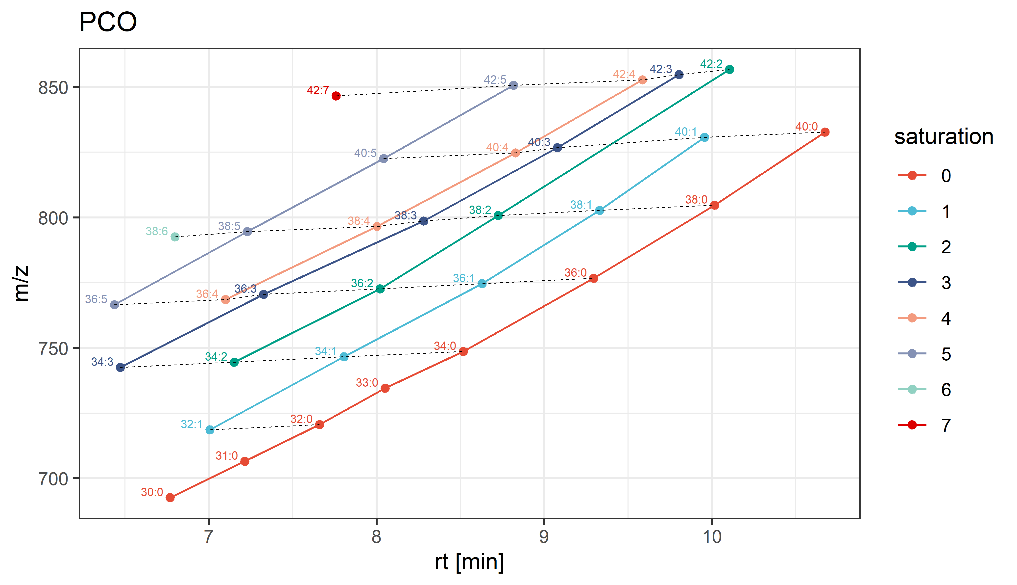

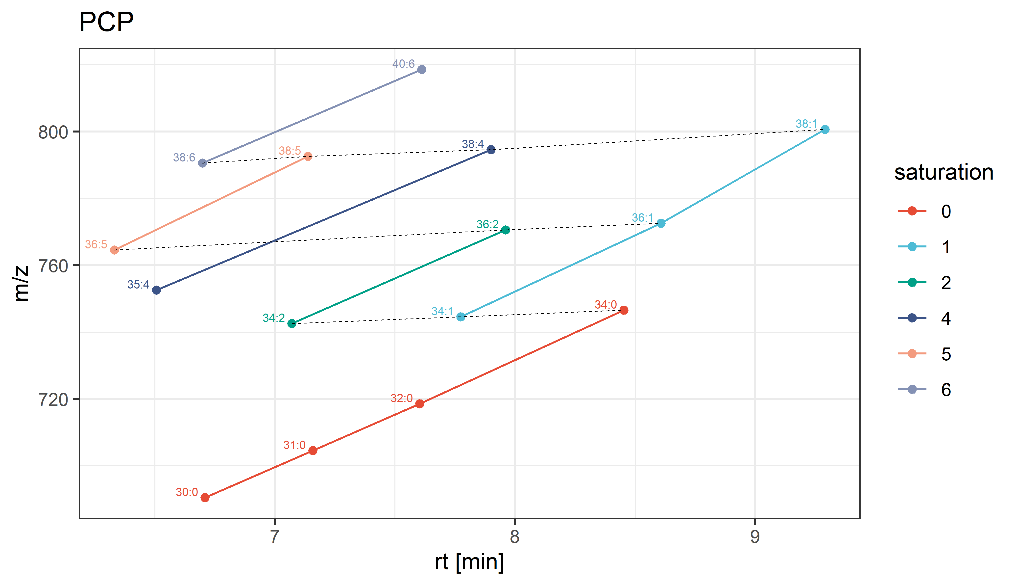
**
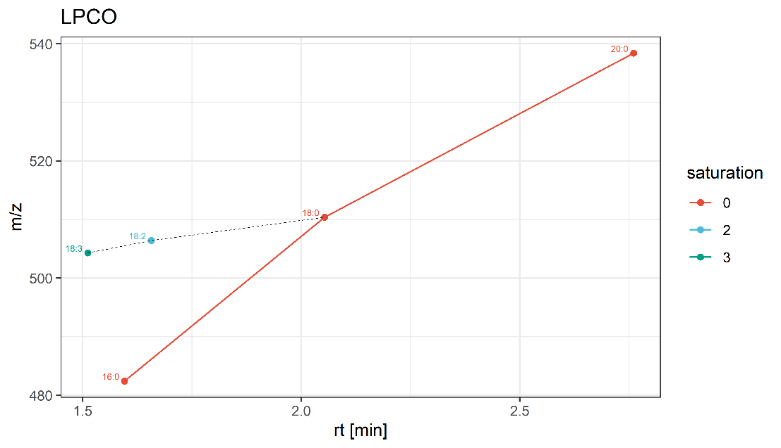

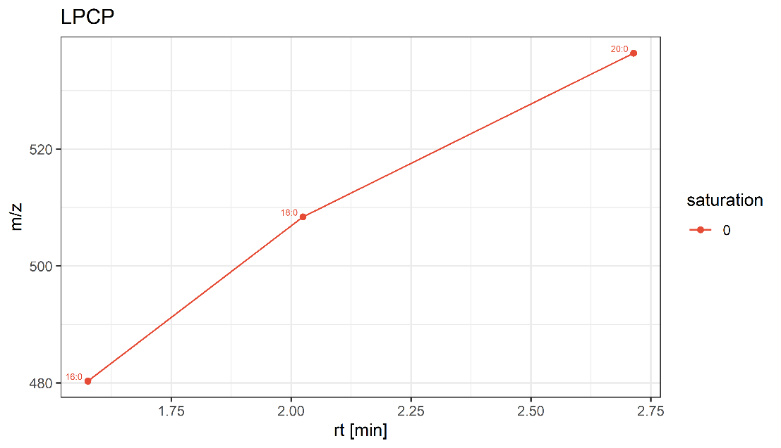


**Fig. S2** Accuracy assessment for SRM 1950 - "Metabolites in Frozen Human Plasma" (number of independently prepared replicates: n=10). Values are presented as normalized coverage equivalents at the mean (dots) and standard deviation (error bars) of measurements, overlaid onto the consensus mean value (blue line) and uncertainty (95% coverage-green region, 99% coverage-red region). These plots are a direct export from the LipidQC software.

**Cholesteryl esters (CE) and cholesterol Ceramides (CER)**

**Hexosylceramides (HexCer) Diacylglycerols (DAG)**

**Free fatty acids (FFA) Lysophosphatidylethanolamines (LPE)**

**Lysophosphatidylcholines (LPC) including plasmanyls (LPC O-) and plasmalogens (LPC P-)**

**Phosphatidylcholines (PC) including plasmanyls (PC O-) and plasmalogens (PC P-)**

**Phosphatidylethanolamines (PE) including plasmanyls (PE O-) and plasmalogens (PE P-)**

**Phosphatidylinositols (PI) and phosphatidylglycerols (PG)**

**Sphingomyelins (SM)**

**Triacylglycerols (TAG)**

**Fig. S3** PCA (A) and OPLS-DA (B) analysis showing the effect of the dysfunctional mutation of the *ABCG2* gene (such as p.Q141K and other mutations with the same dysfunctional effect) on the lipidome of all patients compared by wildtype (WT), heterozygous (HET) or homozygous (HOM) gene inheritance.

**A) B)**

**Fig. S4 –** Validation of the OPLS-DA models from Figure 1 B (A) and Figure 1 C (B) based on permutation test performed with 999 permutations.

**A) B)**

**
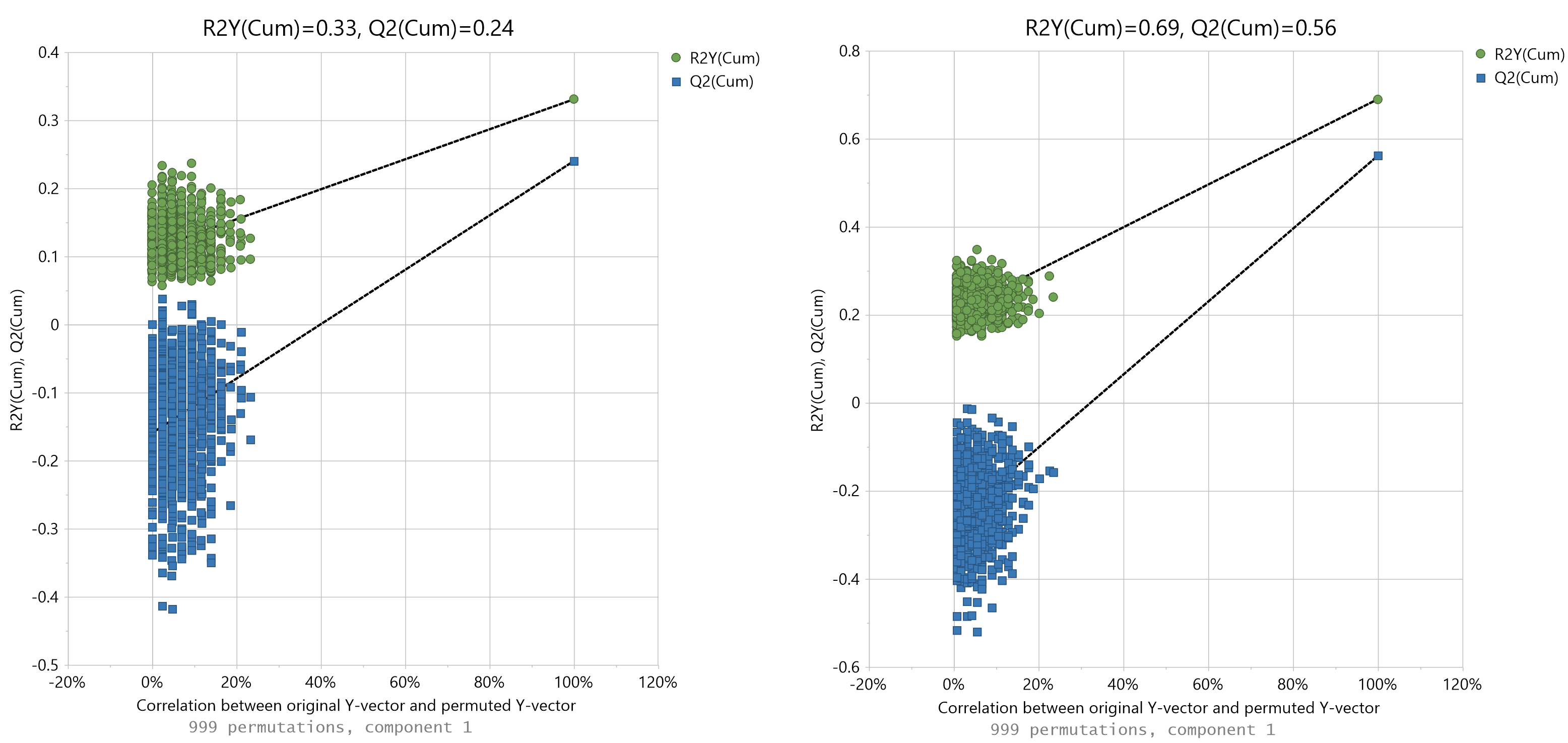
**

**Fig. S5** Overview of lipid networks based on hyperuricemia (HUA), gout and age of onset/detection ≤/>40 years and urate-lowering therapy (T0/T1) versus controls. The size of the nodes was adjusted according to the maximum -log p-value for each individual network.

**
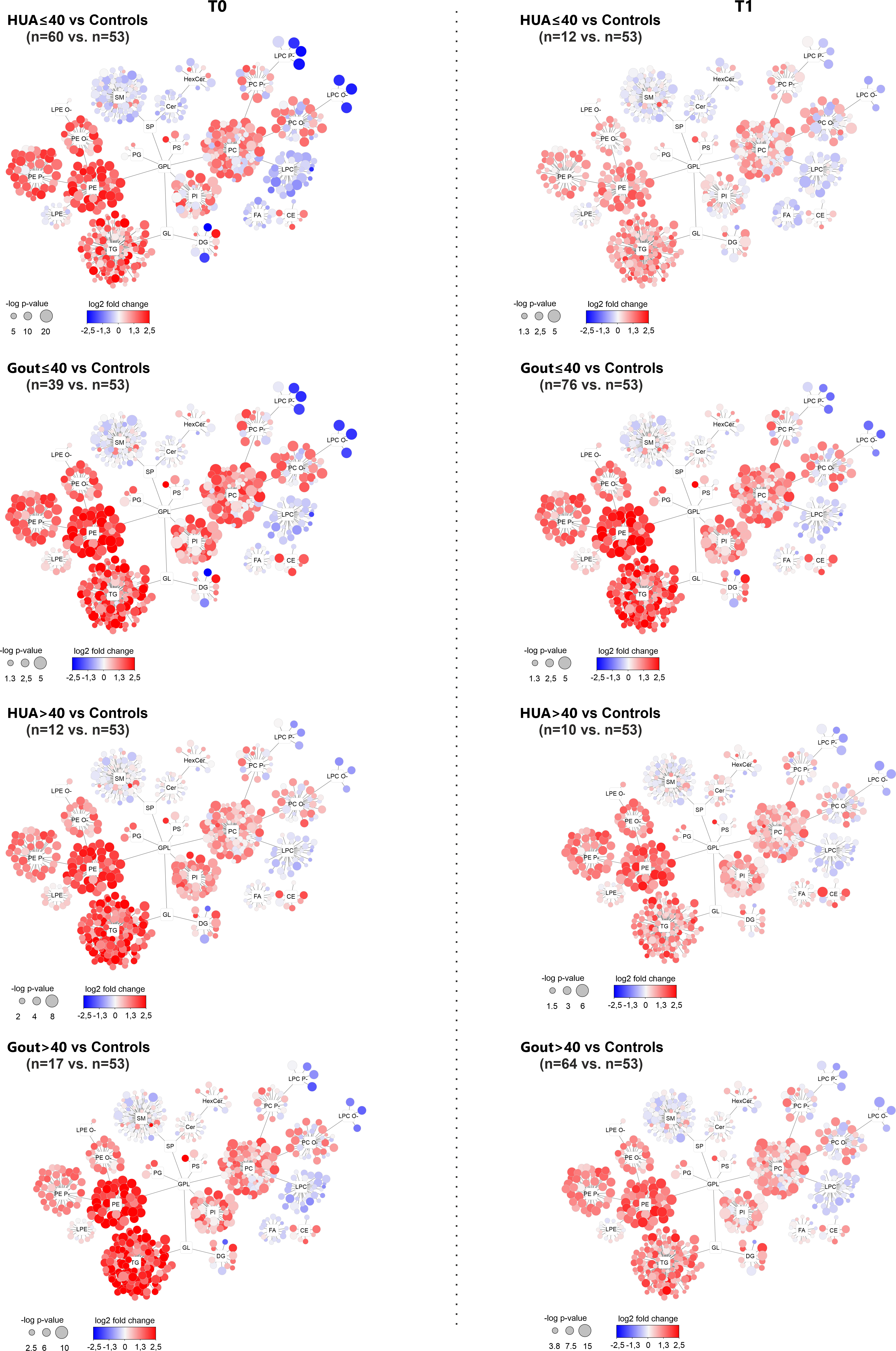
**

**Fig. S6** Influence of sex on the changes in lipidome comparing hyperuricemia (HUA) and gout versus controls. The size of the nodes was adjusted according to the maximum -log p-value for each individual network.

**
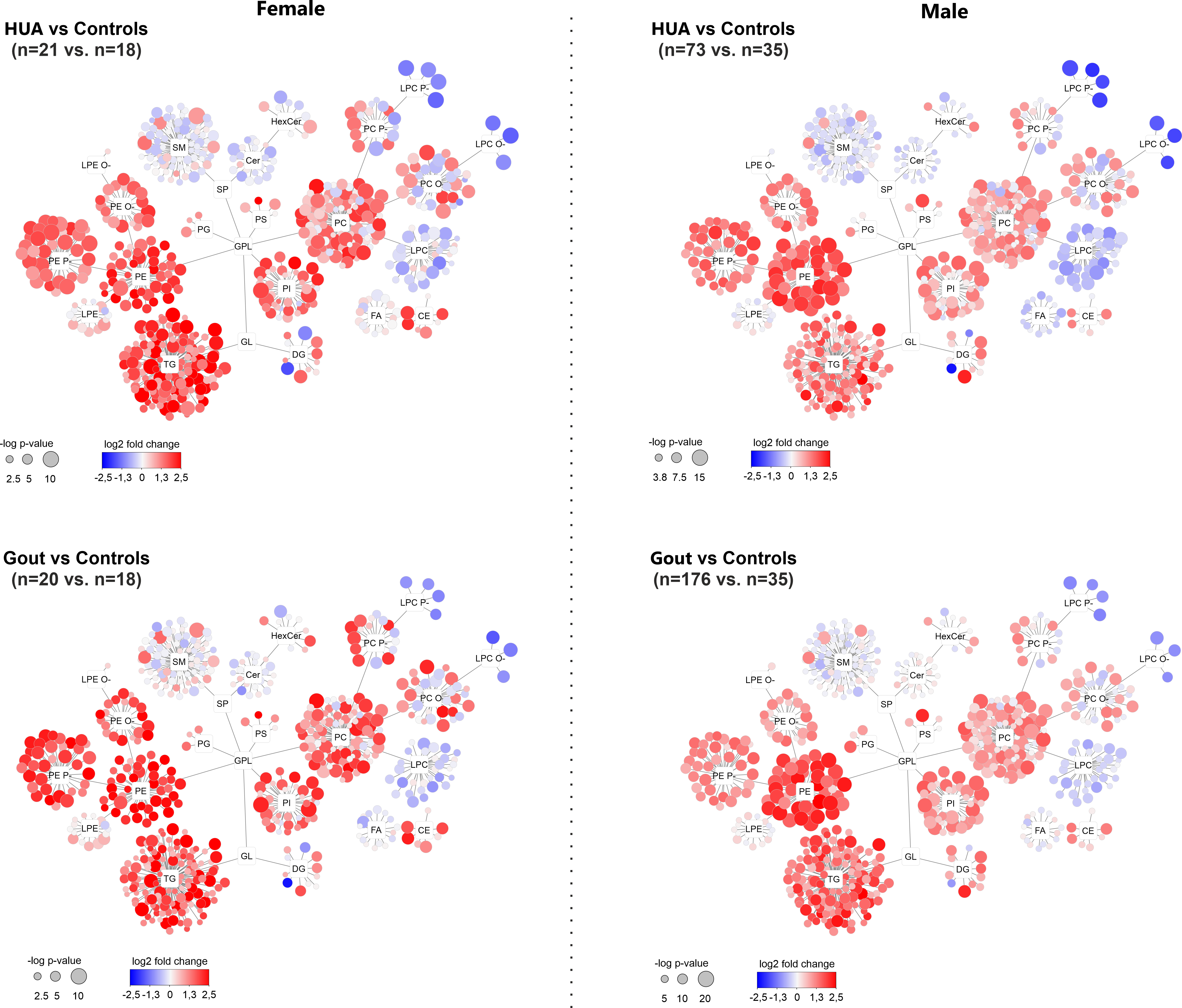
**

**Fig. S7** Influence of age on the changes in lipidome comparing hyperuricemia (HUA) and gout versus all controls and age-matched controls. The size of the nodes was adjusted according to the maximum -log p-value for each individual network.


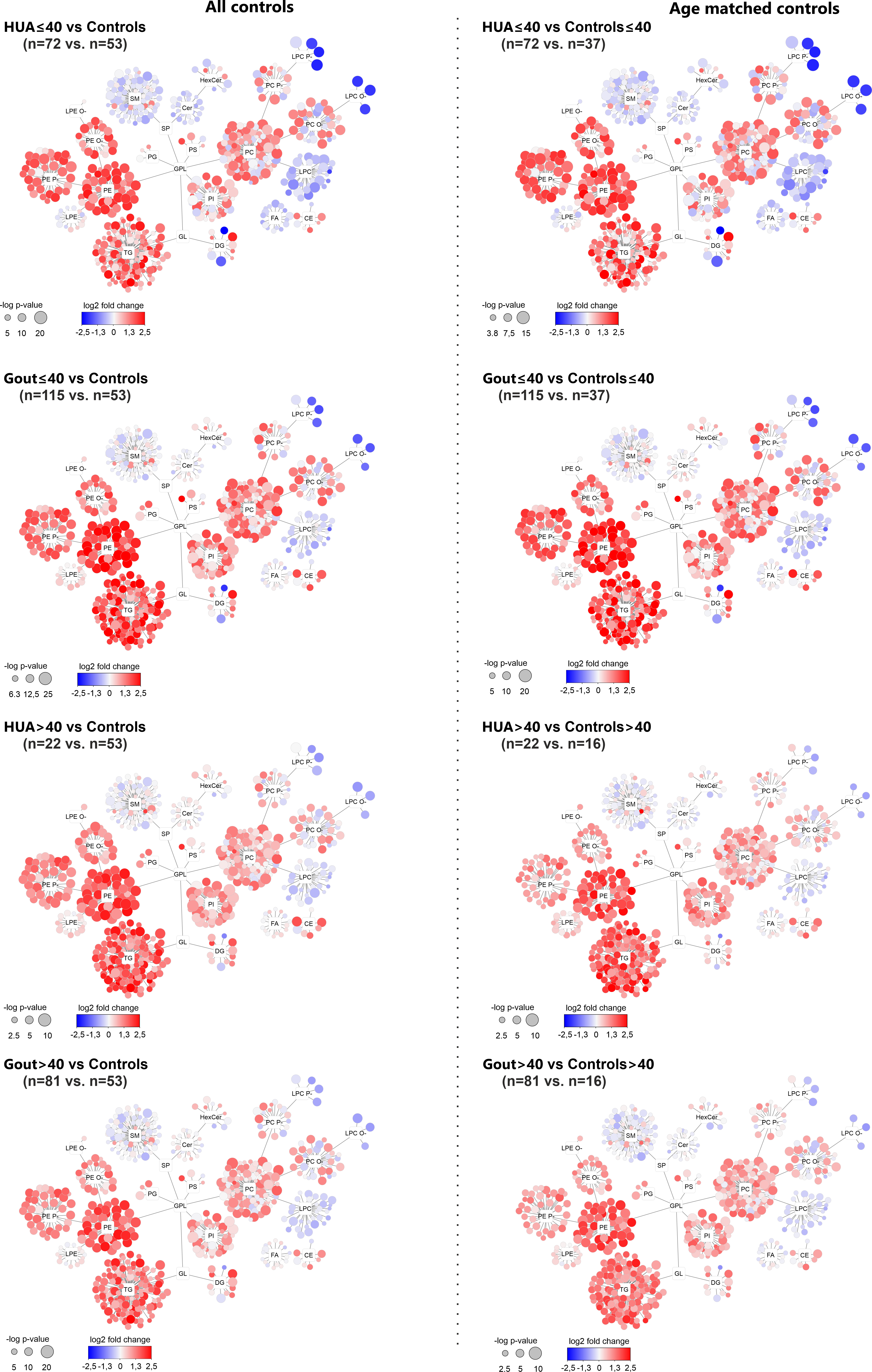

Supplement: Supplementary file 1 — Additional file 1: Supplementary File. Detailed characterization of the sample preparation, LC-MS lipidomic analysis, data processing and semiquantification of lipids. Fig. S1. Lipid patterns plotted separately for each lipid class. Fig. S2. Accuracy assessment for SRM 1950 - "Metabolites in Frozen Human Plasma" (number of independently prepared replicates: n=10). Fig. S3. PCA (A) and OPLS-DA (B) analysis showing the effect of the dysfunctional mutation of the ABCG2 gene (such as p.Q141K and other mutations with the same dysfunctional effect) on the lipidome of all patients compared by wildtype (WT), heterozygous (HET) or homozygous (HOM) gene inheritance. Fig. S4. Validation of the OPLS-DA models from Figure 1 B (A) and Figure 1 C (B) based on permutation test performed with 999 permutations. Fig. S5. Overview of lipid networks based on hyperuricemia (HUA), gout and age of onset/detection ≤/>40 years and urate-lowering therapy (T0/T1) versus controls. Fig. S6. Influence of sex on the changes in lipidome comparing hyperuricemia (HUA) and gout versus controls. Fig. S7. Influence of age on the changes in lipidome comparing hyperuricemia (HUA) and gout versus all controls and age-matched controls. [file 13075_2023_3204_MOESM1_ESM.docx]
